# Effects of Different Combinations of Phytochemical-Rich Fruits and Vegetables on Chronic Disease Risk Markers and Gene Expression Changes: Insights from the MiBLEND Study, a Randomized Trial

**DOI:** 10.3390/antiox13080915

**Published:** 2024-07-29

**Authors:** Julia N. DeBenedictis, Courtney Murrell, Duncan Hauser, Marcel van Herwijnen, Bart Elen, Theo M. de Kok, Simone G. van Breda

**Affiliations:** 1 Toxicogenomics Department, GROW School of Oncology & Reproduction, Faculty of Health, Medicine & Life Sciences, Maastricht University, 6229 ER Maastricht, The Netherlands; 2 Flemish Institute for Technological Research (VITO), 2400 Mol, Belgium

**Keywords:** phytochemicals, prevention, disease risk, synergy, fruit, vegetables, transcriptomics, DNA damage, antioxidant, nutrigenomics

## Abstract

Adequate fruit and vegetable (F and V) intake, as recommended by the World Health Organization (over 400 g/day), is linked to reduced chronic disease risk. However, human intervention trials, especially with whole F and V and in complex combinations, are lacking. The MiBlend Study explored the effects of various phytochemical-rich F and V combinations on chronic disease risk markers, phytochemical absorption, and gene expression in blood. This randomized cross-over study involved participants consuming two of seven different F and V blends for 2 weeks (450 g/day), following a 2-week low F and V intake period (50 g/day). Each blend represented major phytochemical classes (flavonoids, anthocyanins, carotenoids, and glucosinolates) or combinations thereof. Markers of chronic disease risk, including DNA damage, oxidative stress, and retinal microvasculature, were measured. Increasing F and V intake significantly improved plasma antioxidant capacity, DNA damage protection, and retinal arteriolar dilation. Flavonoid-rich, carotenoid-rich, and complex blends notably reduced DNA damage susceptibility. Anthocyanin-rich and carotenoid-rich interventions were most effective in boosting antioxidant capacity, while blends high in flavonoids, especially combined with anthocyanins, significantly improved retinal microvasculature. Gene expression analysis revealed changes in DNA repair, signal transduction, and transcription processes, indicating mechanisms for these health benefits. The study suggests specific F and V blends can provide targeted health improvements, emphasizing the importance of both overall F and V intake and the specific phytochemical composition for personalized preventive strategies.

## 1. Introduction

The role of dietary habits in chronic disease development has long been a subject of scientific inquiry. Epidemiological evidence consistently suggests that increased consumption of fruits and vegetables (F and V) is associated with a reduced risk of chronic diseases such as cardiovascular disease, diabetes, and certain types of cancer [1,2]. A 2017 meta-analysis estimated that 5.6 million premature deaths in 2013 worldwide may be attributable to insufficient F and V intake [3]. Despite this evidence, most Western populations still fail to meet the WHO-recommended minimum intake of >400 g of F and V per day (excluding starchy tubers) [4]. Only 10% of Americans and 12% of Europeans meet recommended intake levels, with the lowest quintile in the Netherlands consuming only 50 g per day [5,6,7].

One suggested explanation for the association between adequate F and V consumption and reduced chronic disease development lies in the rich content of phytochemicals present in these foods. Phytochemicals are naturally occurring, non-caloric bioactive compounds found in plants. Apart from their role in providing F and V their vibrant colors, these compounds are believed to function as natural defenses for plants against environmental stressors and hold significant potential for promoting human health. To date, approximately 20,000 phytochemicals have been characterized, and they exist in complex mixtures within plants [8]. This naturally occurring complex of phytochemicals can affect multiple mechanisms of action, resulting in biological effects, which are equal to, lesser than (in case of antagonism), or greater than (in the case of synergism) than the cumulative effect of the isolated compounds [9].

The ability of phytochemicals to mitigate chronic disease risk lies in their capacity to reduce established etiologies such as oxidative stress, inflammation, and impaired cellular signaling [10,11]. Their major mode of reducing oxidative stress is by scavenging and neutralizing free radicals in the body. This antioxidant activity has relevant implications for cancer development. Neutralizing free radicals prevents damage to cellular organelles and macromolecules like DNA and proteins, preventing the promotion of carcinogenesis [12].

Beyond their antioxidant potential, phytochemicals, particularly polyphenols found in foods like apples, berries, and green tea, have demonstrated their capacity in regulating various stages of carcinogenesis. They can influence cellular processes associated with anti-tumor activities such as proliferation, apoptosis, cell cycle progression, inflammation, angiogenesis, and the prevention of invasion and metastasis, as evidenced by numerous in vitro and animal models [13].

Sulfur-containing isothiocyanates present in cruciferous vegetables have been recognized for their influence on Phase I and Phase II biotransformation enzymes and their inhibitory effects on histone deacetylases (HDAC) [14,15]. For instance, sulforaphane, found in broccoli, induces Phase II detoxification enzymes like quinine reductase and glutathione transferase while inhibiting cytochrome P450 2E1, an enzyme associated with carcinogen activation in animal models [16,17]. Some human studies have yielded results consistent with these mechanisms and their role in cancer prevention. In a human pilot study, consumption of a single serving (68 g) of broccoli sprouts led to the inhibition of HDAC activity in peripheral blood mononuclear cells within 3–6 h following ingestion [15]. This action, led by isothiocyanates like sulforaphane in broccoli, is believed to provide access to genes otherwise restricted by cancer pathology, facilitating cell cycle arrest and apoptosis. Another human dietary study showed that fortifying processed red meat with plant extracts rich in phytochemicals resulted in a significant reduction in the excretion of N-nitroso compounds associated with processed red meat consumption and colorectal cancer development [18].

Phytochemical-rich foods have also been studied for their role in promoting cardiovascular health. Their consumption has been demonstrated to improve blood pressure and blood lipids, inhibit LDL oxidation, and reduce platelet aggregation, reducing the risk of cardiovascular disease, atherosclerosis, and abnormal clot precipitating events, respectively [9,19,20,21]. Polyphenol-rich foods have also been shown in *in vivo* animal models to induce enzymes that produce nitric oxide, like glutathione endothelial nitric oxide synthase (NOS) and inducible NOS, which is necessary for the regulation of blood vessel function, blood pressure regulation, and immune responses [22]. Additionally, a randomized prospective study with 27 healthy participants found that food rich in a type of polyphenol called catechins led to reduced vascular tone, increased peripheral vascular dilation via activation of the nitric oxide system, and augmented the vasodilator response to ischemia [23].

In addition to their demonstrated role in preventing cancer and promoting cardiovascular health, phytochemicals are also known to modulate our gene expression. These compounds may target transcription factors, directly affecting DNA transcription and potentially leading to many of these downstream physiological alterations. After consuming foods high in phytochemicals, studies have shown gene-expression changes in humans related to processes involving gene-transcription regulation, cellular-signaling cascades, immune response, lipid and glucose metabolism, apoptosis, and platelet aggregation and activation [11,24,25]. However, there are limited studies indicating gene expression changes along with corresponding changes in phenotypic biomarkers.

While nearly all of the research that looks at the composition of F and V consumption or dietary patterns and chronic disease risk comes from observational or prospective cohort studies, investigations into phytochemicals have traditionally centered around single compounds at non-physiological concentrations or the examination of individual F and V, typically through *in vitro* and animal models [7]. While these studies have provided valuable insights into the mechanisms of individual compounds, there is a compelling need to expand our exploration of the impact of phytochemicals within the context of whole foods, particularly in human intervention studies [9].

While there is a limited number of human studies investigating different combinations of F and V, they indicate the potential of various combinations of F and V in modulating disease risk markers and gene expression [26,27,28]. However, there is a scarcity of studies that investigate both gene expression and relevant disease risk markers simultaneously. Gene expression changes offer insights into the molecular mechanisms affected by nutrition intervention. Understanding how genes are regulated provides a mechanistic understanding of the observed phenotypic outcomes. Phenotypic changes serve as tangible, measurable outcomes, validating the relevance of specific gene-expression changes. Their inclusion strengthens the connection between molecular alterations and observable health effects, so the inclusion of both provides a more comprehensive and holistic understanding of an intervention’s effect. Additionally, the optimal F and V blend for protecting the body against chronic diseases remains uncertain. Therefore, further human studies exploring combinations of phytochemicals, known for their diverse mechanisms of action, are needed to evaluate their impact on both gene expression and disease risk markers.

To bridge these gaps, the MiBLEND study (Bioactives Lead to ENhanced Defense) was created. The first aim of the MiBLEND study is to investigate the effects of increasing F and V consumption from a low (50 g/day) intake to a recommended level (450 g/day) on markers associated with chronic disease risk and gene-expression changes in healthy volunteers. The second aim is to determine the effect of different blends of F and V, each rich in specific phytochemical classes and varying in complexity. These blends are provided in a ready-to-consume form, having already been weighed, blended, bottled, and undergone low-temperature pascalization in order to preserve high levels of bioactive ingredients and prevent spoilage [29]. The outcomes of this study include changes in DNA damage susceptibility, oxidative stress, retinal microvasculature, absorption of bioactive compounds, and gene expression. By analyzing these different phytochemical combinations, the study aims to elucidate the intricate mechanisms by which they interact within the human body, potentially unlocking new insights into the prevention and management of chronic diseases.

## 2. Materials and Methods

### 2.1. Study Design

**Figure 1:**
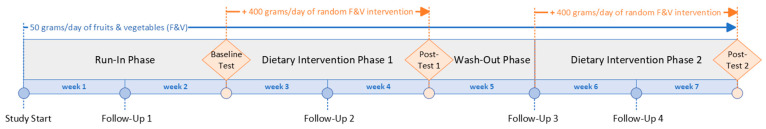
MiBlend study timeline.

This study employs a randomized crossover design to explore the impact of various combinations of F and Vs, along with an overall increase in consumption, on biomarkers of chronic diseases in healthy human volunteers (Figure 1). The study spanned 7 weeks. Each participant started with a 2-week run-in period, followed by a 2-week intervention phase, a 1-week washout period, and, finally, another 2-week intervention phase. The run-in period was designed to standardize everyone’s F and V intake to the same, low level of 50 g per day so that baseline intake levels were uniform across all participants, and so that the effects of increasing F and V intake at recommended levels during intervention phases (450 g per day) were more likely to be identifiable within a 2-week duration. Participants were randomized to receive two out of seven distinct nutrition interventions, and the 1-week washout phase was added to mitigate any potential carry-over effect from the first intervention into the second intervention phase, a duration validated by a prior study [18].

Throughout the study, participants consumed 50 g of F and V per day at their own discretion, but during intervention phases, participants added on their randomly assigned blend of 400 g of F and V. Participants kept a digital food diary (MyFitnessPal) that was regularly checked by a Registered Dietitian Nutritionist. Phytochemical-rich food consumption was controlled throughout the study, including a limitation of two cups of coffee or tea per day. Consumption of dietary supplements, fruit or vegetable juices made from real F and V, red wine, and green tea were not permitted. If participants were alcohol drinkers, high-risk drinking was restricted. Female participants were limited to a maximum of two alcoholic drinks per day, no more than twice per week, and males were limited to three drinks per day, no more than three times per week.

On both baseline and post-intervention test days, participants arrived in the morning at the research institute to undergo a fasting blood draw and to deliver urine collected over the previous 24 h. Additionally, a photograph of their right eye fundus, as well as height and weight measurements, was taken.

In addition to test days, participants also came in for weekly follow-up visits to meet with the study coordinator to check in and exchange study materials. On visits when dietary interventions were distributed, 1 week’s worth of the randomized blend was provided at a time, and at the subsequent visit, participants would return their emptied bottles to the study coordinator to check for compliance.

### 2.2. Dietary Interventions

Seven different blends of F and V were evaluated in this study. The dietary interventions were prepared as previously described [29]. Briefly, the blends were prepared by MiFood (Venlo, The Netherlands), which weighed, blended, bottled, and pascalized (using high-pressure, low-heat technologies to sterilize) the F and V listed in Table 1. Blends 1–4 contained a selection of F and V, resulting in an overrepresentation of a specific class of phytochemicals. Blends 5–7 consisted of a combination of the four different classes overrepresented in Blends 1–4, with increasing complexity.

To promote an evenly distributed consumption of the blends throughout the day, each blend was portioned into small bottles containing 100 g of the mixture, and participants were provided four bottles per day to consume. All blends were prepared in a single batch according to the methods described in DeBenedictis et al. (2023) and kept frozen at −18 °C [29]. The study coordinator would remove 1 week’s worth (28 bottles) of a blend at a time from frozen storage and thaw them at 4 °C for 48 h prior to distribution. Participants were required to keep their bottles in cold storage as long as possible throughout their intervention phases. The nutrient composition of each dietary intervention can be found in Table 1. The phytochemical composition of each blend based on the content of the measured raw materials is provided in Supplementary Figure S1.

### 2.3. Participants

Participants were recruited via online advertising and paper flyers in Maastricht, The Netherlands. The study was approved by the local Medical Ethics Review Committee of the Maastricht University Medical Centre+ (MUMC+) (registration number: NL66118.068.18), registered at the International Trial Registry Platform (ICTRP) under identifier: NL7358, and conducted according to the guidelines laid down in the Declaration of Helsinki. At least 1 week of contemplation time was provided between when participants learned of the study and signed the informed consent documents. All participants provided written informed consent followed by a medical questionnaire before study commencement. At study start, participants were instructed on how to log their daily intake in a food diary. Food diaries were analyzed for accuracy and compliance weekly.

All participants were required to be healthy subjects between 18 and 60 years old and with a BMI range of 18.5–27 kg/m^2^. Exclusion criteria were alcohol abuse up to 6 months prior to the study, current smokers and ex-smokers up to 3 months prior to the study, current presence or symptoms related to any diseases of the gastrointestinal tract, kidney, liver, heart or lungs, or diseases related to the endocrine or metabolic systems, HIV infection, hepatitis, or anemia. Other reasons for exclusion were pregnancy, having used antibiotics 3 months prior to the study, taking medications other than contraceptives, the presence of a food allergy relevant to the dietary intervention, following a vegetarian or vegan diet, high activity levels (>8 h per week of vigorous activity), or current participants of other intervention studies. Participation was monitored under the care of a Registered Dietitian Nutritionist for the duration of the study.

### 2.4. Test Days

On test days, the study coordinator reviewed the participant’s food diary and collected their 24 h urine flask(s). These were weighed, homogenized, and aliquoted into freezer-safe Eppendorf tubes and stored at −20 °C until later 8-OHdG, 8-isoprostane, and Vitamin C analysis. Then, the participant’s height and weight were measured. The baseline test day weight was used to calculate the participant’s BMI (kg/m^2^). Next, a fasting blood sample was collected in EDTA-coated tubes. Blood was immediately aliquoted for RNA and superoxide preservation, and the rest was stored on ice until later processing and storage at −80 °C, or delivery to the MUMC+ clinical diagnostic lab for blood composition analysis. Finally, a trained study technician took a photo of the fundus of the participant’s right eye using a Canon CR-2 non-mydriatic retina camera (Serial number: S/N 103138). These images were saved for later microvasculature analysis. Test day procedures were always performed in the same order for each visit.

### 2.5. DNA Damage Markers

#### 2.5.1. DNA Strand Breaks

The alkaline comet assay was performed on isolated lymphocytes on the same day as test-day blood collection. Participant lymphocytes at a concentration of 1 × 10^6^ cells/mL were exposed to 25 µM H_2_O_2_ (Merck, Darmstadt, Germany) and immediately incubated along with an unexposed control sample for an hour at 37 °C. These samples were mounted to microscope slides in triplicate with 0.65% low melting point agarose (Sigma Aldrich, St. Louis, MO, USA) and then lysed in pH 10 lysis buffer (Buffer: 2.5 M NaCl (Sigma Aldrich), 100 mM EDTA (Sigma–Aldrich), 10 mM Tris (Sigma Life Sciences, Darmstadt, Germany), and 250 mM NaOH (Sigma–Aldrich). Added same day: DMSO (Sigma–Aldrich) and Triton X-100 (Sigma–Aldrich)) to a final concentration of 10% and 1%, respectively, at 4 °C overnight. The slides were unwound in electrophoresis buffer (Milli-Q water, 300 mM NaOH, and 1 mM EDTA) for 40 min and electrophoresed for 20 min at 1 V/cm. The slides were removed, washed with 1xPBS, and then dried with ethanol. Slides were stored in opaque slide holders at 4 °C until stained with 10 µg/mL ethidium bromide (Cleaver Scientific, Rugby, UK), covered with coverslips, and, an hour later, imaged on a BIO-TEK Cytation 3 imaging reader with Gen5 software version 3.05. These images were saved, and Comet Assay IV software version 3.11 (Instem) was used to score comets. Fifty comets from each participant sample were assessed, and the mean of all participant triplicate samples’ % Tail DNA and Tail Moment data were recorded. The same researcher performed all comet assay laboratory work and comet scoring to prevent experimental and interrater variability.

#### 2.5.2. Oxidized Purines

The group that had Blend 7 was selected for 8-Hydroxydeoxyguanosine (8-OHdG) analysis as this was the most complex blend, and we hypothesized that it might, therefore, affect multiple mechanisms of action related to reducing oxidative stress, potentially leading to a physiological reduction in oxidized purines. Two AssayGenie 8-OHdG ELISA kits, which each include a 96-well plate, and all necessary reagents were used. Urine samples, which had been stored at −80 °C, were thawed and diluted with the provided dilution buffer. Plate standards and reagents were prepared according to the kit instructions. Each well was washed with wash buffer twice before 50 µL of participant urine samples and standards were added to the wells in duplicate. Then, 50 µL of Biotin-labelled antibody working solution were added to each well, and then the plate was incubated for 45 min at 37 °C. Each well was then washed three times, then 100 µL of HRP-Streptavidin Conjugate working solution were added, and then the plate was incubated for 30 min at 37 °C. Finally, each well was washed five times, 90 µL of TMB substrate were added to each well, and then the plate was incubated for 10–20 min in the dark (until a gradient appeared in standard wells) at 37 °C. The reaction was stopped with 50 µL of the STOP solution in the same order as the TMB substrate, which was added to the wells. Absorbance was read by a multiplate reader (CLARIOstar Plus, BMG LABTECH, Ortenberg, Germany), and the absorbance was read at 450 nm. Absorbance values were corrected with the standard blank, and a standard curve plotted (R^2^ = 0.98) was used to calculate the 8-OHdG concentration from the averaged duplicate absorbance values of the participant samples. These concentration values were then corrected with their dilution factor and creatinine concentrations, which were measured in separately thawed urine aliquots by the MUMC+ Clinical Diagnostic Laboratory.

### 2.6. Oxidative Stress in Blood and Plasma

#### 2.6.1. Antioxidant Capacity

Participant plasma samples for analysis were thawed from storage at −80 °C. Phosphate buffer was prepared with sodium phosphate monobasic (Merck, Darmstadt, Germany) dissolved in Milli-Q water and brought to a pH of 7.4 using 1M NaOH (Merck, Darmstadt, Germany). The phosphate buffer was prepared weekly. The radical solution was prepared by dissolving 2,2′-azinobis (3-ethylbenzothialozine-6-sulfonic acid) di-ammonium salt (ABTS) (Sigma, Sigma–Aldrich, Steinheim, Germany, CAT: A1888) in phosphate buffer. Then, 2,2′-azobis-(2-amidinopropane)(HCl)2 (ABAP) (Polysciences, Inc., Warrington, PA, USA, CAT: 08963) was dissolved in a beaker with phosphate buffer. These two solutions were combined in a flask, and 50 mL of phosphate buffer were added to reach an end volume of 70 mL. The flask was placed in a 70 °C water bath for 10 min. The absorbance was checked at 8, 9, and 10 min at 734 nm (iMark microplate reader, BioRad, Hercules, CA, USA). The optimal absorbance is 0.7 + 0.02. When the optimal absorbance was reached, the radical solution was placed on ice. The radical solution was brought back to 37 °C using a water bath before beginning the experiment. The radical solution and the Trolox dilutions were prepared daily. The Trolox dilutions were made by dissolving (±)-6-hydroxy-2,5,7,8-tetramethyl-chroman-2-carboxylic acid (Trolox) (Aldrich, Sigma–Aldrich, CAT: 238813) in 39.95 mL of phosphate buffer, resulting in a 1 mMol/L solution. This solution was mixed with phosphate buffer, and then seven Trolox dilutions were made from this solution and used as the standards. Phosphate buffer alone was used as a blank control, which was later subtracted from sample absorbance values. The absorbance of radical solution (37 °C) was used to denote t = 0. Then, 280 µL of the radical solution were mixed with 15 µL of participant plasma sample (diluted 1:2 with phosphate buffer) and added to a 96-well plate with a multichannel pipette, and the absorbance was immediately read at 734 nm. The plate was then incubated at 37 °C for 5 min, and the absorbance was measured at t = 5. All samples were measured in triplicate. The difference in absorbance from t = 5 to t = 0 provided the change in solvent absorbance, and this was subtracted from the change in Trolox absorbance from t = 5 to t = 0. The regression coefficient was calculated from the calculated change in Trolox absorbance to create the calibration curve (average R^2^ = 0.99) and used to calculate sample TEAC values.

#### 2.6.2. Superoxide Scavenging

Immediately after blood collection on test days, 250 µL of whole blood were removed from EDTA blood-collection tubes into a freezer-safe Eppendorf tube and mixed with 250 µL of CMH (1-hydroxy-3-methoxycarbonyl-2,2,5,5-tetramethylpyrrolidine), a superoxide-specific spin-trapping agent. (Noxygen Science Transfer and Diagnostic GmBH, Elzach, Germany). The spin-trapping agent was prepared as previously described in large aliquoted batches and stored at −20 °C until being thawed just prior to use [31]. The blood-and-spin-trap mixture was then flash-frozen with liquid nitrogen and then stored at −80 °C until analysis. Levels of superoxide were measured by electron-spin resonance (ESR) spectroscopy. The frozen blood samples were thawed on ice for 50 min before analysis, and 100 µL of the sample were drawn up into glass capillary tubes (Brand, Wertheim, Germany). ESR spectroscopy was performed at 21 °C using an EMX X-band spectrometer (Bruker, Billerica, MA, USA) and commercially available software (version 2.11, Bruker Win EPR System) as previously described [32]. Spectra were quantified by double integration of peak surface as previously described [31].

### 2.7. Retinal Microvasculature

#### 2.7.1. Vessel Calibre (CRAE, CRVE, and AVR)

All test-day fundus images of a participant were measured together to ensure analysis consistency using MONA-REVA software (3.0.0 software, VITO, Mol, Belgium). The six largest arterioles and venules were outlined and, their caliber and AVR were calculated by the software. The means for each participant test day were recorded. The same researcher performed or validated all vessel-caliber analyses to prevent interrater variability.

#### 2.7.2. Vessel Branching and Tortuosity

Tortuosity index and branching counts have been determined for each fundus image. The software determines both measures automatically using vessel network segmentation, but these vessel networks were manually checked for their accuracy. Analysis was executed in accordance with the MONA-REVA 3.0.0. User’s Guide (VITO).

### 2.8. Phytochemicals in Plasma and Urine

#### 2.8.1. Total Polyphenols

Participant plasma was taken from −80 °C storage and thawed on ice. To pre-treat the samples, plasma was mixed with 0.75 mol/L metaphosphoric acid (MPA) (Sigma–Aldrich). This was vortexed for 3 minutes, and then aliquoted and mixed with 1 mol/L HCl (VWR). This solution was vortexed for 2 min and then incubated for 30 min at 37 °C (Eppendorf ThermoMixer). Then, 2 mol/L NaOH were added and vortexed for 2 min. After 30 min of incubation at 37 °C, 0.75 mol/L MPA were added and vortexed for 2 min. The samples were then centrifuged at 20,000× *g* for 10 min and the supernatant was collected in a separate tube. The supernatant was extracted from the pellet by adding acetone:water (1:1) (Sigma) and centrifuging at 2700× *g* for 10 min. The supernatant was added to the previously collected supernatant. To measure the total polyphenol content in the samples, the samples were mixed with Folin–Ciocalteu reagent (FCR) (Sigma) (diluted 10× with distilled water). The mixture was then incubated for 5 min and then sodium carbonate (Merck) was added. This mixture was added to the wells of the microplate. The plate was then incubated for 90 min at room temperature, and then the absorbance was measured via a microplate reader (CLARIOstar Plus, BMG LABTECH) at 725 nm. The standard used was gallic acid (Merck) (100, 50, 25, 12.5, 6.25 μg/mL) dissolved in 50% methanol (Honeywell). A standard curve was plotted (average R^2^ = 0.99). The resulting concentration of the samples was then expressed as mg/g of gallic acid equivalents (mg GAE/g). All samples were measured in triplicate.

#### 2.8.2. Carotenoids

Carotenoid measurements were taken from plasma thawed from −80 °C storage. Carotenoid concentrations were measured according to instructions provided by ClinRep HPLC Complete Kit (RECIPE Chemicals + Instruments GmbH, Munich, Germany) and as described elsewhere [33]. The column oven was set to 60 °C, injection volume was 100 µL, wavelength of 450 nm, flow rate was 0.6 mL/min, Mobile Phase A of 97.5% methanol: 2.5% water, and Mobile Phase B was isopropanol.

#### 2.8.3. Vitamin C

A redox–titration method was used to measure Vitamin C concentrations in participant urine. For the titration, 2,6-dichlorophenolindophenol (DCPIP) was used as a coloring reagent. The DCPIP solution was made by combining DCPIP (Merck) with 1.46 mMol sodium acetate (Sigma–Aldrich) and Milli-Q water in an Erlenmeyer flask. Sodium citrate buffer was made by adding 62.64 mMol sodium acetate and 240 mL of Milli-Q water and adjusted to pH of 3.5 with 20% phosphoric acid (VWR Chemicals BDH, Tilburg, The Netherlands). Vitamin C solution was made by combining 0.09 mMol ascorbic acid into 100 mL of Milli-Q water to make a 1.6 mg/mL stock. A serial dilution was made of ascorbic acid solution, including a blank control. Then, 85 µL of sodium citrate buffer and 170 µL DCPIP were added to the standard (85 µL) wells. Participant urine samples, which had been stored at −80 °C, were thawed and homogenized gently with a table vortex. Then, 300 µL of sample were mixed with 555 µL DCPIP and added to the wells in triplicate (340 µL total per well). The plate was then covered in foil and brought to the CLARIOstar multi-plate reader, which read absorbance at 520 nm for 1:30 min at room temperature. Absorbance values were corrected with the standard blank and a standard curve plotted (average R^2^ = 0.99) and used to calculate the Vitamin C concentration from the averaged triplicate absorbance values of the participant samples. These concentration values were then corrected with their creatinine concentrations, measured from urine samples by the MUMC+ clinical diagnostic lab. Due to the known degradation of Vitamin C in frozen storage [29], the number of months that each sample was stored in frozen storage was included as a covariate in the statistical model for this variable.

### 2.9. Gene Expression Analysis

#### 2.9.1. RNA Isolation

Whole blood was removed from EDTA collection tubes immediately after venipuncture on test days and added to freezer-safe Eppendorf tubes containing 2:1 RNAlater (Ambion, Austin, TX, USA). Samples were stored at −80 °C until thawed on ice for RNA isolation. All test days from a participant were isolated in the same batch. While the RNAlater solution preserved the RNA in our samples, the salt content was not compatible with the extraction steps of this kit, so the following removal steps were followed to remove the RNAlater from solution. Then, 900 µL of the samples were centrifuged for 1 min at 5000× *g* and at room temperature. The supernatant was removed and discarded. Also, 150 µL of room temperature 1xPBS were added and mixed into the pellet gently via a table vortex. QIAzol lysis reagent (QIAGEN) was added to the samples 3:1 and mixed with the vortex. Then, 300 µL of chloroform (VWR Chemicals BDH) were added and vortexed immediately after each sample. All samples were manually shaken for 30 s and then incubated at room temperature for three minutes. Then, the samples were centrifuged for 15 min at 1200× *g* and at 4 °C. The resulting supernatant was transferred to new 2 mL Eppendorf tubes, and then 1000 µL ethanol were added to each sample and gently vortexed. RNA isolation was then performed using the Directzol RNA microprep kit (ZY-R2052, Zymo, Irvine, CA, USA), following the manufacturer’s instructions. The isolated RNA was stored at −20 °C until library prep was performed. Sample quality and concentration control were ensured with a Qubit Fluorometer (Introgen Life Technologies, Carlsbad, CA, USA) and Bioanalyzer (Agilent Technologies, Santa Clara, CA, USA). If any samples did not meet quality control standards (RIN > 7), they were reisolated and reassessed.

#### 2.9.2. Library Prep and Sequencing

Purified RNA samples were prepared for sequencing using the NextFlex Rapid Directional RNA kit 2.0 with ribonaut HMR ribo-depletion and unique dial indices (Perkin Elmer, Waltham, MA, USA). All RNA samples were checked again for concentration with the Qubit Fluorometer and for library size with the D1000 Screentape and D1000 High Sensitivity tapes (Agilent 2200 TapeStation, Boston, MA, USA). All samples were normalized according to their concentration and library size for an RNA input of 100 ng. The library prep was performed in nine batches of about 48 samples, each with a Zephyr-calibrated robotic system (Perkin Elmer) monitored by a trained technician. Sequencing was performed with three 200-cycle S4 flow cells (Illumina, San Diego, CA, USA) of around 150 samples each. Samples were de-multiplexed and reviewed for quality. All participant test-day samples were kept together within batches.

#### 2.9.3. DATA Pre-Processing

The raw data produced by sequencing were demultiplexed and converted from BCL files to fastQ format, trimmed with fastp-0.20.0, and mapped with STAR-2.7.9a to the human reference genome (GRCh38). Reads were then quantified with RSEM-1.3.1 to produce a table of raw gene counts. Additional digital cleaning was done to remove ribosomal RNA missed by ribo-depletion and globin-related genes [34]. The cleaned output was then used for differential expression gene analysis.

#### 2.9.4. Differentially Expressed Genes (DEG) and Pathway Analysis

All count data were loaded into R (version 4.3.0). All data with “NA” were replaced with 0. Outliers, contributing more than 20% to the overall variance along a principle component, were removed. Samples with a total read count below 3 million were removed. Remaining participant samples lacking a matched baseline or post-test sample were removed. A DGE object was created with the package “edgeR” (3.38.4) with sample meta-data, count data, and gene information for down-stream analysis. Raw counts were then converted to counts per million (CPM) and log-CPM for figure representation. Genes with low expression levels were removed according to previously described methods [35]. Gene expression was then normalized for the entire dataset using the trimmed mean of M-values [36]. This function is integrated in edgeR and creates normalization factors that are used as scaling factors for the library sizes. MDS plots were then created to observe the unsupervised clustering of the samples and determine which factors should be included in the statistical model. Unsupervised MDS clustering showed evidence of clustering around the factors: “sex”, “age_bin”, “batch”, “wbc_count”, and “covid”. Participant age was discretized into the closest age bins separated by 5 years (e.g., “20”, “25”, “30”, etc) to provide the value in “age_bin”. “Batch” represents the sequencing batch that the samples were pooled in. “Wbc_count” represents the binned WBC count values. Actual WBC count values of 4 mL participants’ blood on that TD were rounded to the nearest whole number, and samples that contained high or low values were binned into a “High” or “Low” group. Finally, “covid” represents the samples that were collected before and after the COVID-19 pandemic began.

Therefore, the design for the model was:

design <- model.matrix(~0 + subset + sex + age_bin + batch + wbc_count + covid)

The correlation coefficient of participant identifier “PCode” was then calculated using the “statmod” package (1.5.0). A design matrix and contrasts were then created with the “limma” package (3.52.3). The contrast matrices, which determine the comparisons being made in the model, were specified as follows:
contr.matrix <- makeContrasts(
D0vsD1 = subsetPostTestD1 − subsetBaselineD1, 
D0vsD2 = subsetPostTestD2 − subsetBaselineD2, 
D0vsD3 = subsetPostTestD3 − subsetBaselineD3, 
D0vsD4 = subsetPostTestD4 − subsetBaselineD4, 
D0vsD5 = subsetPostTestD5 − subsetBaselineD5, 
D0vsD6 = subsetPostTestD6 − subsetBaselineD6, 
D0vsD7 = subsetPostTestD7 − subsetBaselineD7, 
AllDIs = (subsetPostTestD1 + subsetPostTestD2 + subsetPostTestD3 + subsetPostTestD4 + subsetPostTestD5 + subsetPostTestD6 + subsetPostTestD7)/7 − (subsetBaselineD1 + subsetBaselineD2 + subsetBaselineD3 + subsetBaselineD4 + subsetBaselineD5 + subsetBaselineD6 + subsetBaselineD7)/7, 
levels = colnames(design)) 


DEG analysis began with 389 samples, and outlier samples identified as being more than 20% different than another sample were removed. Samples with a total read count below 3 million were removed. From a starting total of 46,664 genes, additional genes were removed for having less than 1 CPM.

Heteroscedasticity was then removed from the count data using the voom function in limma by applying precision weights to the data, making limma’s computational methods appropriate for sequencing data. The model was then fitted to the expression values for each gene, and then empirical Bayes moderation was conducted by borrowing information across all genes to obtain more precise estimates of gene-wise variability [37]. A summary of DEGs was created for each intervention group and for the combination of all baseline and post-test samples. Code and output of this analysis can be found at https://github.com/jndeben/miblend, accessed on 29 May 2024. DEGs with an FDR *p*-value < 0.05 for the comparison of all baseline samples versus all post-intervention samples were input in Reactome’s web-analysis tool [38]. The pathways above an adjusted (FDR) *p*-value threshold of *p* < 0.05 were recorded. Pathways related to disease pathways were filtered out of presented tables and figures due to their irrelevance within the context of a healthy study population undergoing a lifestyle intervention but are still included in the full Supplementary Tables showing all results.

#### 2.9.5. Gene Set Enrichment Analysis (GSEA)

To uncover biological insights that DEG analysis could not identify by intervention group, GSEA was employed to identify coordinated changes in gene expression associated with specific biological processes. This method provides a more holistic understanding of the underlying biology, which can identify subtle changes missed by DEG analysis (where individual gene changes might not be statistically significant, but collectively represent meaningful biological changes) and helps to generate hypotheses to guide further investigations. The ranked gene lists for each comparison group resulting from the DEG analysis have been analyzed with R using the “clusterProfiler” (4.4.4), “fgsea” (1.22.0), and “ReactomePA” (1.40.0) packages. The genes in each comparison were ranked by their t values, which contain the directionality (up- or down-regulation) and the magnitude (or intensity) of the gene-expression differences between the groups relative to the variability within the samples. The Reactome gene set enrichment pathways provided the predefined sets of genes that GSEA utilized to associate the expression changes to specific biological pathways. Enrichment analysis was then performed, which determined whether the members of a gene set are enriched at the top (or bottom) of a ranked gene list, reflecting the degree of overrepresentation or underrepresentation of the gene set in the ranked list. This was done using a statistical algorithm that calculated an enrichment score for each gene set. Then, permutation testing was performed to assess the significance of enrichment scores. The gene labels were randomly permuted, and the enrichment analysis was performed again to obtain a null distribution of enrichment scores. The observed enrichment scores were then compared to the null distribution to calculate an empirical value, and then multiple testing correction was applied. The *p*-value threshold was defined as *p* < 0.05 adjusted with the Benjamini-Hochberg (FDR) method. Genes with a fold-change near zero are effectively down-weighted to insignificance. Pathways related to Disease pathways were filtered out of presented tables and figures due to their irrelevance within the context of a healthy study population undergoing a lifestyle intervention but are still included in the full Supplementary Tables showing all results. Code and output for this analysis can be found at https://github.com/jndeben/miblend, accessed 29 May 2024.

### 2.10. Sample Size Calculation and Randomization

To ensure sufficient statistical power to detect effects in the gene-expression analyses (the endpoint which requires the largest group size) between the dietary groups, the power calculation tool PowerAtlas [39] was applied to previous human-intervention data [10,11] to calculate required group sizes. An effect size of 20% and a false discovery rate (FDR) corrected *p*-value of <0.05, using the Benjamini–Hochberg method, will be considered for correction of multiple testing (maximal 5% of significant findings could be false positive). Aiming at a statistical power of 80% and an (multiple testing corrected) alpha level of 0.05, a group size of 40 individuals per intervention should be sufficient to detect effects on the transcriptomic level. Since this power calculation is based on a previous intervention study, which was not as strictly controlled as the study suggested here will be, statistical power can be expected to be even higher at the calculated group sizes.

Randomization was calculated using computer-generated random numbers in Microsoft Excel (2016) described previously [40]. The random allocation sequence, participant enrollment, and participant assignment were performed by the coordinating investigator. Participant assignment occurred 48 h prior to the first (baseline) test day to allow for the preparation of the intervention product for subsequent distribution. Participants were blinded to the ingredients contained in the dietary interventions, as the bottles were only labeled with the intervention number, and ingredients were blended into a homogenous mixture.

### 2.11. Statistical Analysis

The statistical method utilized in this study for all endpoints aside from gene-expression analysis is a linear mixed model, or, more specifically, a marginal model for repeated measures performed in IBM SPSS Statistics (Version 26). This method accounts for missing values (dropouts), as well as repeated measures with the same participant, allows for the inclusion of fixed effects (e.g., age, sex, BMI), and can determine if the cross-over design created an order effect. We utilized an unstructured covariance type, and all tests included the covariates “Age” and “BMI” and the factor “Sex”. When analyzing the effect of period or the three test days on the dependent variables, “period” was also included. When analyzing the effect of dietary intervention group on the outcomes, first “Group”, “Period”, and “Period × Groups” were included as fixed effects to determine if there was an order effect. If no order effect was observed, the factors “Groups × Period” and “Period” were removed from the analysis. The analysis was run with least significant differences (LSD) corrections, and multiple testing was corrected for with the FDR. Both the *p*-values and the measure of the FDR (*q*-values) associated with each *p*-value are presented, and a threshold < 0.05 determines significance. If certain fixed effects were found to be significant in the model, these were further explored, such as with a correlational analysis of covariates (continuous variables). Post-hoc tests for significant factors included an independent *t*-test (parametric data; equal variances) or Mann–Whitney U test (non-parametric data; equal variances). Seven participant samples were excluded from the final analysis as they did not meet quality criteria for statistical analyses. Final group sizes for each assay are reported in Supplementary Table S1.

## 3. Results

### 3.1. Participants

**Figure 2:**
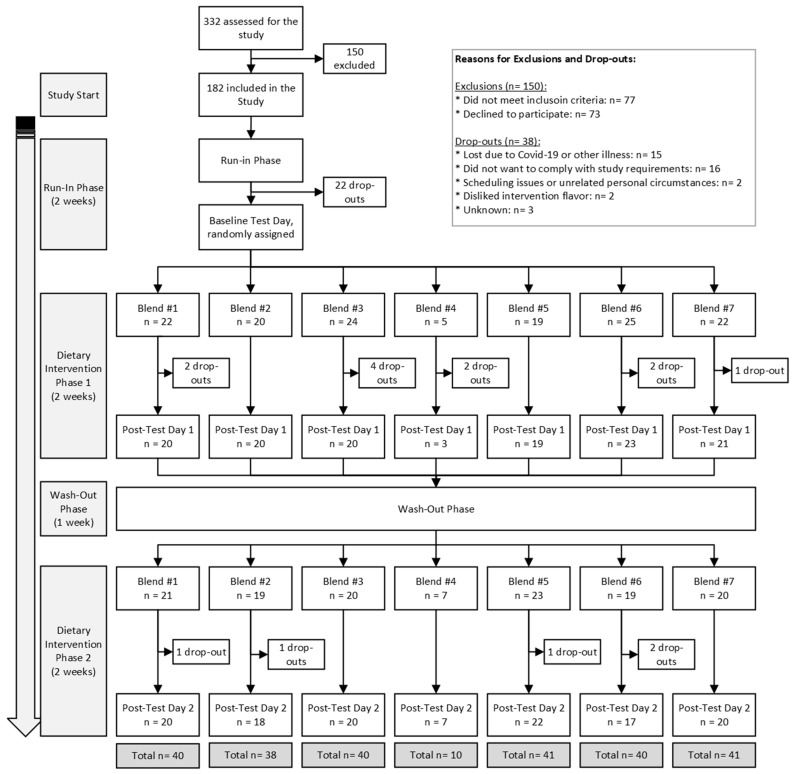
MiBLEND Study results with number of participants included, excluded, dropped out, and total completed interventions.

In total, 182 participants were included in the study, and 146 participants completed the study between March 2019 and May 2022. The trial ended due to achieving sufficient power for each intervention group. Most dropouts were related to illness or being unable to adhere to study guidelines. A flowchart of the study’s participant inclusion, exclusion, and drop-out distribution is presented in Figure 2. The fewer completions of Blend 4 were due to its poor likability/tolerance from participants. To prevent unnecessary dropouts, if a participant could not tolerate Blend 4 after first tasting it, they were immediately re-randomized to a new blend.

The mean sex distribution of the participants was 27% male and 73% female. The average age was 27 + 10 years (min: 18, max: 59), and the mean BMI was 22.7 ± 2.2 kg/m^2^. There were no significant differences between intervention groups for age or BMI. Sex was significantly different between groups, but this difference disappeared when the group who had Blend 4, which is underpowered, was removed from the analysis. Table 2 shows the age, sex, and BMI distribution.

There was a statistically significant increase in body weight at both post-intervention test days compared to baseline (*p* < 0.05). The average baseline weight was 67.46 ± 0.66 kg, and the average weight at Post-tests 1 and 2 was 67.85 + 0.66 kg and 68.06 + 0.66 kg, respectively. This is an average increase of 0.39 kg at Post-test 1 and 0.60 kg at Post-test 2 compared to baseline (Table 2). Weight at Post-test 1 compared to Post-test 2 was not significantly different.

Overall, intervention phases were marked by a statistically significant increased intake of calories, carbohydrates, sugars, and fiber compared to baseline (*q* < 0.05 for all comparisons) (Table 3). This amounted to an average increase of 114 kcal, 38 g of carbohydrate, 20 g of sugars, and 11 g of fiber during intervention phases. The participant fiber intake, which was well below optimal levels at baseline and wash-out phases, reached or nearly reached the dietary recommendations during the intervention phases (recommended intake was 25–35 g/day based on sex and age).

### 3.2. DNA Damage Markers

#### 3.2.1. DNA Strand Breaks

**Figure 3:**
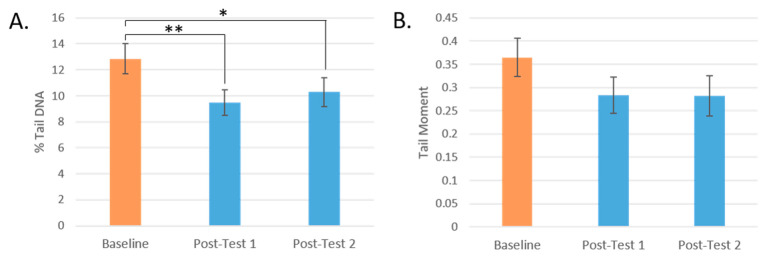
Changes in DNA strand breaks by test day. (**A**): % DNA in Tail by test day. (**B**): Tail Moment by test day. (* = *p* < 0.05, ** = *p* < 0.005. Mean values are presented and the error bars represent the SEM).

At Post-test 1, the percentage of DNA breaks found in the comet tails significantly decreased by 26%, from 12.85% to 9.49% (*p* = 0.002), and by 20% to 10.28% at Post-test 2 (*p* = 0.045) compared to baseline. The tail moment decreased by 22% from 0.37 at baseline to 0.28 at Post-test 1 and Post-test 2 (Figure 3).

**Figure 4:**
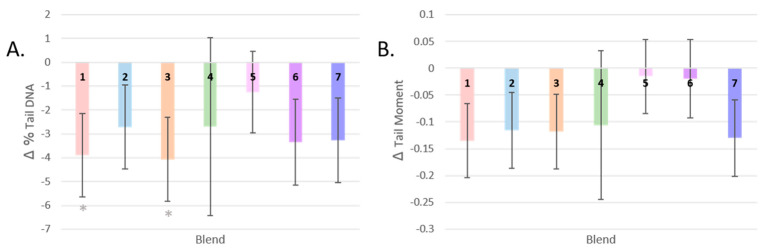
Changes in DNA strand breaks by dietary intervention. (**A**): Change in % tail DNA after each dietary intervention. (**B**): Change in tail moment after each dietary intervention. (* = *p* < 0.05; Gray asterisks represent *p*-values after LSD correction. Mean values are presented and the error bars represent the SEM).

While all groups showed a mean reduction in DNA damage, participants who consumed Blend 3 and Blend 1 showed the greatest reduction in total percentage of DNA strand breaks (by 30% and 31%, respectively) (Figure 4A). These were the interventions with the highest level of carotenoids and total flavonoids, respectively. These changes post-intervention were significant only before FDR correction (*p* = 0.021, *q* = 0.147, and *p* = 0.027, *q* = 0.095, respectively).

The post-intervention tail moment measurements of participants who consumed Blends 5 and 6 did not differ much from baseline, and the largest change in mean from baseline was seen in participants who consumed Blend 1, the intervention highest in total flavonoids (−0.124, a reduction in Tail Moment by 34%, *p* = 0.068) (Figure 4B).

**Table 2:** MiBLEND study results. Values by test day are reported in the first three columns. Post-test values by dietary intervention are reported in Columns 4–10. Values reported are the estimated means after statistical analysis with the standard error. Reported significance is in comparison to matched baseline values, AUC = area under the curve, DEG = differentially expressed genes, GSEA = gene set enrichment analysis. * = *p* < 0.05, ** = *p* < 0.005, *** *p* < 0.001. The number of asterisks signify the *p*-values. The black asterisks represent *q*-values, gray asterisks represented the *p*-valuse before FDR correction.

		Baseline	Post-Test 1	Post-Test 2	Blend 1	Blend 2	Blend 3	Blend 4	Blend 5	Blend 6	Blend 7
Participant Characteristics	N	146	126	117	38	37	40	9	40	39	40
	% Female	73%	74%	72%	68%	79%	60%	40%	78%	75%	88%
	Age (yr)	27 ± 10	27 ± 10	27 ± 9	27 ± 9	26 ± 9	27 ± 11	34 ± 13	27 ± 10	25 ± 7	28 ± 9
	BMI (kg/m^2^)	22.7 ± 2.2	22.6 ± 2.2	22.7 ± 2.2	22.6 ± 2.2	22.1 ± 2.5	22.1 ± 2.0	23.3 ± 2.6	22.5 ± 2.1	22.9 ± 2.4	22.6 ± 2.2
DNA Damage	% Tail DNA	12.85 ± 1.15	9.49 ± 0.99 **	10.28 ± 1.11 *	9.04 ± 1.67 *	10.22 ± 1.72	8.86 ± 1.64 *	10.24 ± 3.66	11.68 ± 1.64	9.57 ± 1.75	9.66 ± 1.73
	Tail Moment	0.37 ± 0.04	0.28 ± 0.04	0.28 ± 0.04	0.24 ± 0.07	0.26 ± 0.07	0.25 ± 0.06	0.29 ± 0.15	0.34 ± 0.06	0.34 ± 0.07	0.26 ± 0.07
	8-OH-dG (ng/mmol creatinine)	36.59 ± 4.04									38.13 ± 3.59
Oxidative Stress Markers	TEAC	1154 ± 9	1186 ± 9 ***	1190 ± 9 ***	1187 ± 12 ***	1198 ± 12 ***	1191 ± 11 ***	1209 ± 21 *	1185 ± 12 **	1195 ± 12 ***	1165 ± 12
	Superoxide Height (AU)	3380 ± 171	3636 ± 197	3285 ± 157	3454 ± 254	3170 ± 259	3277 ± 244	3279 ± 446	3666 ± 250	3154 ± 257	3476 ± 251
	Superoxide AUC (×10^5^ AU)	12.04 ± 0.63	13.31 ± 0.79	11.98 ± 0.65	12.93 ± 1.03	12.13 ± 1.05	11.59 ± 1.00	12.27 ± 1.82	13.23 ± 1.02	11.31 ± 1.05	12.87 ± 1.03
Retinal Microvasculature	CRAE (µm)	121.08 ± 3.62	123.88 ± 3.76 ***	124.75 ± 3.79 ***	123.08 ± 3.87 *	123.78 ± 3.86 *	123.39 ± 3.87 *	125.96 ± 4.35 *	124.69 ± 3.86 ***	124.30 ± 3.86 **	123.77 ± 3.86 *
	CRVE (µm)	177.36 ± 5.28	175.52 ± 5.16 *	177.51 ± 5.14	174.45 ± 5.25 *	175.81 ± 5.26	175.81 ± 5.24	177.99 ± 5.75	175.99 ± 5.26	177.77 ± 5.26	176.53 ± 5.27
	AVR	0.67 ± 0.01	0.70 ± 0.01 ***	0.70 ± 0.01 ***	0.70 ± 0.01 ***	0.69 ± 0.01 ***	0.69 ± 0.01 ***	0.70 ± 0.01 *	0.70 ± 0.01 ***	0.69 ± 0.01 ***	0.70 ± 0.01 ***
	Branching	60.54 ± 1.47	59.54 ± 1.48	61.23 ± 1.50	61.77 ± 1.87	59.65 ± 1.90	59.69 ± 1.87	58.40 ± 3.12	60.12 ± 1.87	61.03 ± 1.88	59.60 ± 1.91
	Tortuosity	0.897 ± 0.001	0.897 ± 0.001	0.897 ± 0.001	0.896 ± 0.002	0.897 ± 0.002	0.898 ± 0.002	0.897 ± 0.004	0.896 ± 0.002	0.897 ± 0.002	0.897 ± 0.002
Phytochemicals in Plasma & Urine	Total polyphenols	254 ± 2	253 ± 2	253 ± 2	253 ± 3	252 ± 3	253 ± 3	248 ± 6	255 ± 3	252 ± 3	253 ± 3
	Lutein (nM)	277.6 ± 12.6	311.8 ± 12.9 ***	301.2 ± 13.3 *	292.6 ± 17.7	317.7 ± 18.2 *	322.6 ± 17.4 **	294.8 ± 31.6	293.6 ± 17.8	272.6 ± 17.9	341.4 ± 17.6 ***
	Lycopene (nM)	444.5 ± 20.1	471.1 ± 21.5	458.6 ± 19.9	433.7 ± 28.9	461.9 ± 29.7	475.5 ± 28.3	473.3 ± 52.08	452.1 ± 29.1	480.7 ± 29.1	472.0 ± 28.7
	Alpha-carotene (nM)	128.4 ± 11.8	150.2 ± 11.1 *	151.9 ± 9.3 *	105.1 ± 12.3	106.3 ± 12.5	212.3 ± 12.0 ***	128.3 ± 20.6	112.7 ± 12.4	179.7 ± 12.4 ***	176.3 ± 12.2 ***
	Beta-carotene (nM)	636.8 ± 44.5	737.9 ± 45.9 ***	759.4 ± 42.3 ***	572.0 ± 52.8	568.2 ± 53.8	955.4 ± 51.9 ***	598.4 ± 84.6	621.9 ± 53.2	853.6 ± 53.1 ***	911.8 ± 52.6 ***
	Vitamin C (mg/mmol creatinine)	0.66 ± 0.05	0.73 ± 0.04	0.74 ± 0.06	0.63 ± 0.07	0.69 ± 0.07	0.85 ± 0.07 *	0.89 ± 0.15	0.70 ± 0.07	0.81 ± 0.07 *	0.61 ± 0.07
Gene expression	Significant DEGs	all post-tests vs. baseline = 846			1	2	2	18	0	9	4
	Significant pathways after GSEA	all post-tests vs. baseline = 281			281	76	244	199	291	301	5

**Table 3:** Average daily food diary nutrient results. *** *p* < 0.001. The gray asterisk represented the *p*-values. The black asterisks represent *q*-values.

	Study Phase				Average Blend
Nutrient Type	Baseline/Run-In Phase	Intervention Phase 1	Washout Phase	Intervention Phase 2	
Calories (kcal)	1708.9 ± 40	1817 ± 42 ***	1693 ± 41	1829 ± 43 ***	182
Carbohydrates (g)	206 ± 7	244 ± 8 ***	202 ± 7	239 ± 8 ***	44
Proteins (g)	78 ± 2	76 ± 3	76 ± 2	80 ± 2	3
Fats (g)	66 ± 2	65 ± 2	65 ± 2	65 ± 3	1
Cholesterol (mg)	145 ± 13	134 ± 14	144 ± 13	152 ± 14	0
Sodium (mg)	865 ± 73	861 ± 76	815 ± 75	931 ± 77	41
Sugars (g)	42 ± 2	66 ± 2 ***	41 ± 2	62 ± 2 ***	28
Fibers (g)	13 ± 1	24 ± 1 ***	13 ± 1	23 ± 1 ***	11

#### 3.2.2. Oxidized Purines

Levels of urinary 8-OHdG did not change in participants after Blend 7 compared to baseline (Table 2). The fixed effect of sex was statistically significant (*p* = 0.025). Female baseline levels were higher than men on average, at 38.7 ± 26.2 ng/mL versus 21.6 ± 13.7 ng/mL. However, the change in male and female 8-OHdG excretion levels after two weeks of consuming Blend 7 was similar and not significantly different than their baseline levels. For example, female 8-OHdG excretion increased on average by 2.0 ± 14.4 ng/mL, and male excretion decreased by 1.6 + 1.5 ng/mL. The differences seen in baseline urinary 8-OHdG by sex could be biological or partially attributed to the differences in group size, as there were 35 females and five males (13%) represented in this group.

### 3.3. Oxidative Stress in Blood and Plasma

#### 3.3.1. Antioxidant Capacity

**Figure 5:**
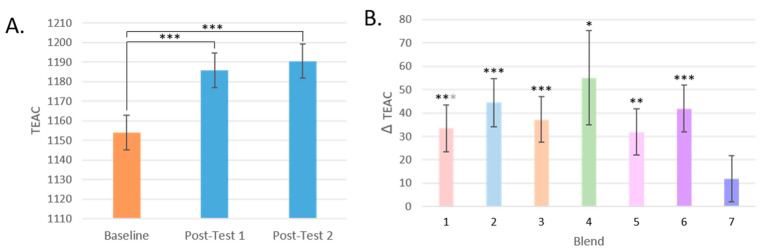
Changes in TEAC. (**A**): TEAC by test day (TD). (**B**): Change in TEAC by dietary intervention group. (* = *p* < 0.05, ** = *p* < 0.005, *** = *p* < 0.001); Black asterisks represent comparisons that are significant after FDR correction; otherwise, if only significant with LSD correction, they are gray. Mean values are presented and the error bars represent the SEM).

The antioxidant capacity of the participants’ plasma significantly increased by 3% after increasing their F and V intake by 400 g/day (*p* < 0.001 for both post-tests). Trolox-equivalent antioxidant capacity (TEAC) levels increased most profoundly in those who consumed Blends 2, 6, and then 3, which are made up of F and V high in anthocyanins, a mixture of F and V high in anthocyanins, carotenoids, and flavonoids, and F and V high in carotenoids (*q* < 0.001 for all comparisons). The next largest increase was found in the group that consumed flavonoid-rich Blend 1, and then finally Blend 5, a blend of flavonoids and anthocyanin-rich F and Vs, and glucosinolate-rich Blend 4. No change in TEAC was found in the plasma of participants who consumed Blend 7, the most complex blend of F and V (Figure 5).

#### 3.3.2. Superoxide Scavenging

Levels of superoxide anion radicals did not change in the whole blood of participants after 2 weeks of increasing F and V intake by 400 g/day (Table 2).

We also investigated changes in superoxide anion radical levels by intervention group. Again, participants belonging to a particular intervention group did not significantly differ in superoxide anion radicals after their intervention compared to baseline. The group that had Blend 6, the blend of catechin, anthocyanin, and carotenoid-rich F and V, had the largest reduction in their group mean, whereas the group that had Blend 5, the blend of catechin and anthocyanin-rich F and V, had the largest increase in their group mean compared with the other intervention groups for both superoxide peak height and AUC (Table 2).

### 3.4. Retinal Microvasculature

#### 3.4.1. Vessel Calibre (CRAE, CRVE, and AVR)

**Figure 6:**
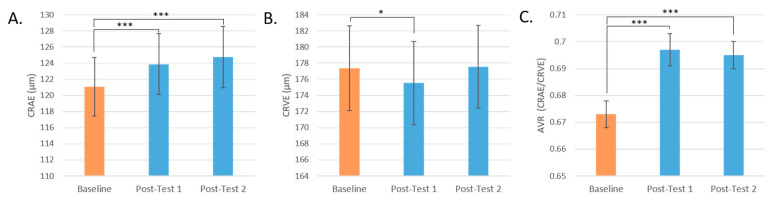
Retinal microvasculature by test day. (**A**): CRAE by Test Day. (**B**): CRVE by Test Day. (**C**): AVR by Test Day. (* = *p* < 0.05, *** = *p* < 0.001. Mean values are presented and the error bars represent the SEM).

Central retinal artery equivalent (CRAE) significantly increased in participants at Post-test 1 and Post-test 2 (*p* < 0.001 for both) by 2.3% and 3.0% (or 2.80 μm ± 0.64 and 3.68 μm ± 0.74), respectively. Central vein equivalent (CRVE) significantly decreased in participants at post-test 1 (*p* = 0.029) by 1% (or 1.85 μm ± 0.83). Arteriolar-to-venular ratio (AVR) increased significantly after all interventions at Post-test 1 and Post-test 2 (*p* < 0.001 for both) (Figure 6).

**Figure 7:**
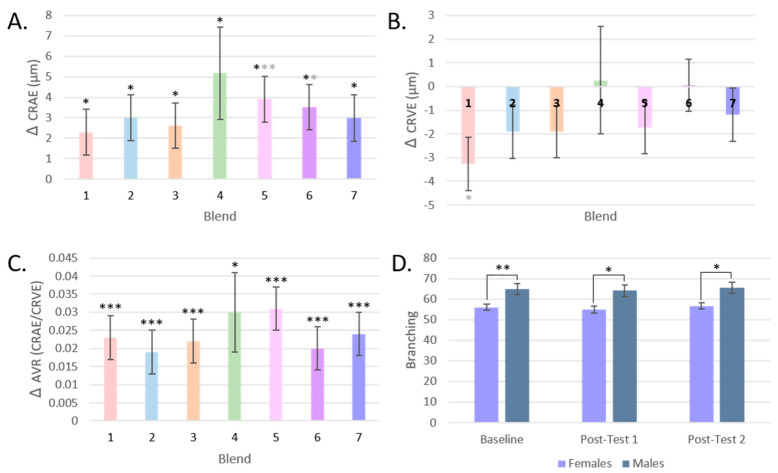
Retinal Microvasculature by dietary intervention. (**A**): Changes in CRAE by intervention group. (**B**): Changes in CRVE by intervention group. (**C**): Change in AVR by intervention group. (**D**): Branching by test day and by sex. (* = *p* < 0.05, ** = *p* < 0.005, *** = *p* < 0.001; Black asterisks represent comparisons that are significant after FDR correction; gray asterisks if only significant with LSD correction. Mean values are presented and the error bars represent the SEM).

The response of CRAE, CRVE, and AVR by the intervention group is represented in Figure 7A–C. CRAE significantly increased for all intervention groups, but most notably for Blend 5 by 3.2% (*p* < 0.001, *q* = 0.004), with a mean increase of 3.91 μm ± 1.11 in retinal arteriolar width. For CRVE, only Blend 1 significantly changed before FDR correction (*p* = 0.018, *q* = 0.126) with a mean decrease of 3.27 μm ± 1.37 or by 1.8% in retinal venular width. AVR, a measure that incorporates both CRAE and CRVE data, significantly changed for all intervention groups. The largest changes were seen in Blend 5 with a mean increase of 0.31 ± 0.006 or by 4.6% (*q* < 0.001), then in Blend 7 with a mean increase of 0.024 ± 0.006 or by 3.6% (*q* < 0.001), and then in Blend 1 with a mean increase of 0.023 ± 0.006 or by 3.4% (*q* < 0.001).

#### 3.4.2. Vessel Branching and Tortuosity

Changes in the branching of the retinal microvasculature of participants were assessed by test day and by intervention group, but no significant differences were found (Table 2). Males did have significantly more branching than females (Figure 7D). Similarly, there was no change in tortuosity post-intervention compared to baseline or by intervention group (Table 2).

### 3.5. Phytochemicals in Plasma and Urine

#### 3.5.1. Total Polyphenols

No significant differences were seen in total polyphenol levels measured in participant plasma by test day or intervention group (Table 2).

#### 3.5.2. Carotenoids

Concentrations of lutein, alpha-carotene, and beta-carotene, but not lycopene, significantly increased in participants’ post-test plasma samples compared to the baseline (Table 2). When testing for an order effect in the carotenoid analysis, a significant interaction was found. Participants who consumed Blend 6 in Intervention Phase 1 had higher mean plasma concentrations of lutein by 41%, alpha-carotene by 37%, and beta-carotene by 32% than those who had Blend 6 at Intervention Phase 2.

There were also differences in carotenoid plasma concentration responses by the intervention group. For lutein, participants who had Blend 7 and Blend 3 had significantly higher plasma concentrations post-intervention compared to baseline by 23% and 17%, respectively (*q* < 0.001; *p* = 0.004, *q* = 0.014, respectively). For lycopene, no significant differences were seen by the intervention group. For alpha-carotene and beta-carotene, groups who had Blends 3, 6, and 7 had higher concentrations post-intervention compared to the baseline (*q* < 0.001 for all) with increases of 34–68%. These results are consistent with the expected concentrations of carotenoids in the blends, with Blend 3 having the highest, followed by Blend 7 for lutein, or followed by Blend 6 and then Blend 7 for alpha- and beta-carotene. The group that had Blend 3 was expected to have the highest concentrations of lycopene, but no significant increase was found in plasma after the intervention.

#### 3.5.3. Vitamin C

There were no significant differences in participants’ Vitamin C levels in post-test urine samples compared to baseline (Table 2). The number of months that the samples were stored at −80 °C was a significant fixed effect in the model (*p* < 0.001), and Vitamin C levels were moderately, negatively associated with time in storage (R^2^ = 0.091) (Supplementary Figure S2).

Excreted Vitamin C concentrations increased significantly in participants who consumed Blend 3, a blend of F and Vs that contains some of the highest expected Vitamin C concentrations of all blends (*p* = 0.007, *q* = 0.049). Blend 6, the blend that has the next highest amount of F and Vs present in Blend 3, significantly increased only before FDR correction (*p* = 0.028, *q* = 0.098) (Table 2).

### 3.6. Gene-Expression Analysis

#### 3.6.1. RNA Isolations

The average RIN value of extracted RNA was 8.4 ± 0.6 across all samples and all 389 samples were considered highly suitable for gene-expression analysis.

#### 3.6.2. Data Pre-Processing

Demultiplexing results can be found at https://github.com/jndeben/miblend, accessed on 29 March 2024. The number of reads from the three flow cells totaled around 30 billion. The balance between samples was also good, with an average of 73.3 million reads per sample (range of 55.6 million to 97.2 million) as a result of normalization and pooling during the library prep and sequencing steps. Quality control reports of trimmed reads show the high quality of the dataset (all bases above Q35). Sequencing depth after trimming ranges between 1 × 10^8^ and 2 × 10^8^ reads. Globin analysis determined that 55.3% ± 9.6% of reads map to 13 selected globins, but three globins, HBA1, HBA1, and HBB, compromised most of these reads.

#### 3.6.3. DEG Analysis

No outlier samples were identified as being more than 20% different than another sample. Six samples were removed because their total read count was below the 3 million read-count threshold. This resulted in 383 total samples. Also, 46,664 genes were removed due to having less than 1 CPM, leaving 10,577 remaining genes.

The voom transformation and heteroscedasticity removal was successful in removing the dependence of the sample variance on expression values in the data. Very few DEGs resulted from DI-group subset analysis, but sufficient DEGs for the effect of all interventions compared to the baseline allowed for a subsequent pathway analysis (Table 4).

The pathways altered after all interventions compared to baseline (i.e., the effect of increasing F and V intake from 50 to 450 g/day) were examined in the Reactome’s pathway analysis web tool. The 10 overrepresented pathways (*q* < 0.05) are shown in Table 5.

The top overrepresented pathway was “HDACs deacetylate histones”, a pathway within the “Chromatin organization” category, under the subgroup “Chromatin modifying enzymes”. The next overrepresented pathway was “RNA Polymerase I promoter opening”, under the category “Gene expression (Transcription)”. Another pathway under “RNA Polymerase I promotor clearance”—“RNA Polymerase I promoter escape”, was overrepresented. Another significant pathway found under the category “Gene expression (Transcription)” was “Epigenetic regulation of gene expression”, particularly “PRC2 methylates histones and DNA”.

Overrepresented pathways under the category “DNA repair” with an FDR *p* < 0.05 include “Depurination”, including its sub-pathways “Recognition and association of DNA glycosylase with site containing an affected purine” and “Cleavage of the damaged purine”. The pathway “RHO GTPase cycle” from the pathway category “Signal Transduction” was also overrepresented. Finally, two pathways under the category “Cell Cycle > Chromosome maintenance” were overrepresented. This includes “Deposition of new CENPA-containing nucleosomes at the centromere” under the sub-category “Nucleosome assembly”. A list of the genes found in these pathways from our DEGs list with their log-fold change and adjusted *p*-values is reported in Supplementary Table S2.

#### 3.6.4. GSEA

**Figure 8:**
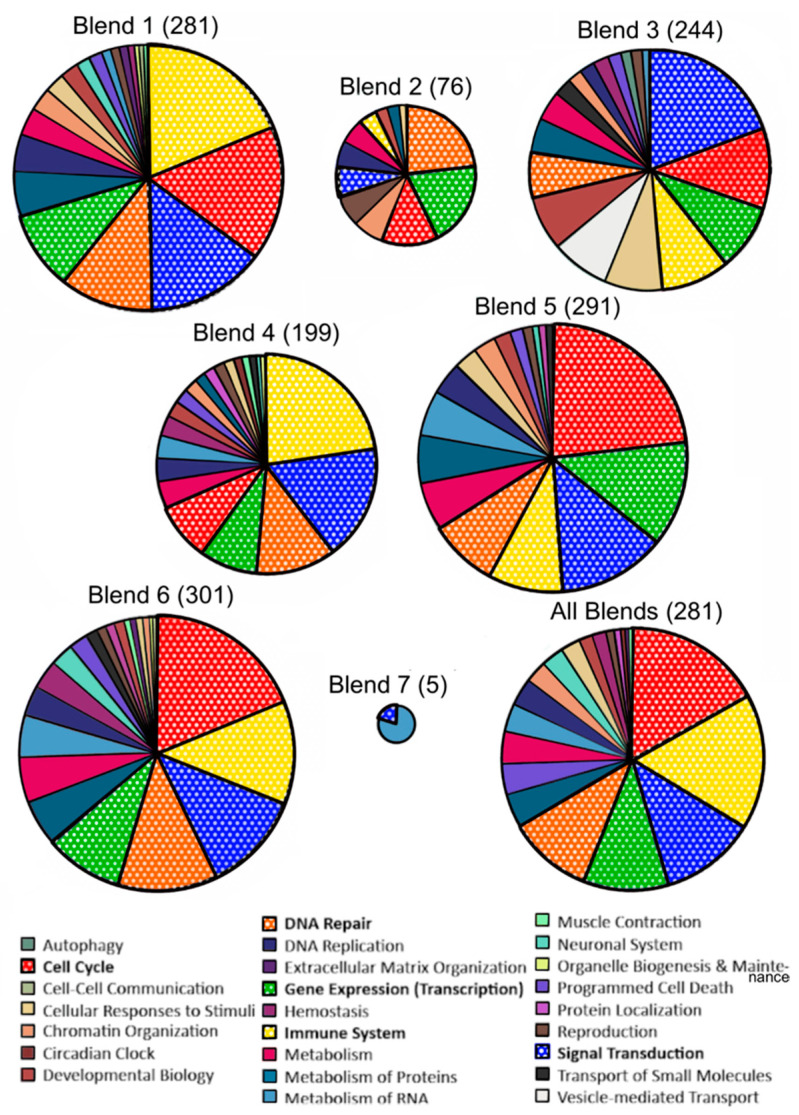
Overview of the proportion of significantly enriched pathways following Gene Set Enrichment Analysis (GSEA) by intervention group. Reactome pathways obtained after GSEA of genes ranked by *t*-value and after FDR correction (*q* < 0.05). Most frequently repeated pathway categories are bolded in the legend and in the pie charts. The size of each pie chart is proportional to the relative number of pathways significant in each comparison.

GSEA was able to identify more relevant biological pathways consistent with the changes in expression levels with each intervention group comparison (Supplementary Tables S3–S9). GSEA was also conducted for all blends (Supplementary Table S10). Most of the pathways altered after the F and V interventions were involved in “Cell cycle”, “DNA Repair”, “Gene Expression (Transcription)”, “Immune system”, and “Signal Transduction” (Figure 8).

## 4. Discussion

In this study, we aimed to establish the impact of different combinations of F and V on biomarkers of DNA damage, oxidative stress, and vascular health, and to identify molecular processes involved in these responses based on gene-expression analyses. The intervention phases comprised 450 g of daily consumption of F and V for 2 weeks, with 400 g encompassing a specific blend of F and V, preceded by a 2-week run-in phase with only 50 g per day of F and V intake. This design facilitates the assessment and comparison of the effects of distinct F and V blends (and their phytochemical components) on markers associated with chronic disease risk. Concurrently, it allows for the investigation of the overall impact of an increase in F and V intake on these outcomes through the analysis of changes across the entire sample set. The main findings were that an overall increase in F and V intake from low to recommended levels improved resistance to *ex vivo*-induced DNA damage (as measured by % Tail DNA), improved plasma antioxidant capacity, increased retinal arteriolar diameter and improved overall retinal vessel caliber, and increased plasma carotenoid concentrations (lutein, alpha-carotene, and beta-carotene). Blend 1 and Blend 3 contributed most to the reduction in DNA damage; all except Blend 7 led to increases in plasma antioxidant capacity; and all improved arteriolar width, whereas Blend 5, Blend 7, and Blend 1 led to the largest improvement in overall microvasculature. Blend 7 led to the largest increase in plasma lutein concentrations. Unsurprisingly, the blends with the largest carotenoid content (Blend 3, Blend 6, and Blend 7) led to the highest concentrations of alpha- and beta-carotene, and the blend with the highest amount of Vitamin C (Blend 3) led to an increase in excreted Vitamin C concentrations. Differentially expressed gene and pathway analyses revealed changes in chromatin organization by HDAC deacytelate histones, and changes in the expression of cell cycle, DNA repair, gene expression, and signal transduction genes from a non-specific increase in F and V intake. GSEA analysis revealed blend-specific alterations in gene-set pathways, but across all blends, most altered pathways came from the same five categories: cell cycle, DNA damage, immune system, signal transduction, and gene expression. While improvements in DNA strand breaks, antioxidant capacity, carotenoid absorption, and retinal microvasculature occurred, no changes were observed in superoxide concentrations in whole blood, excreted 8-OHdG levels, retinal tortuosity or branching, nor plasma lycopene or total polyphenol concentrations.

### 4.1. Protection from Oxidative Stress and Damage

DNA strand breaks were assessed with the comet assay, which stands out as a powerful tool for evaluating the effectiveness of dietary interventions in preventing chronic diseases, particularly cancer [41]. By directly assessing DNA damage at the single-cell level, this assay provides a sensitive measure of genotoxicity [42]. Considering that DNA damage is a key contributor to chronic diseases like cancer, the comet assay becomes instrumental in reflecting how well a person’s cells can withstand damage from oxidative agents. This, in turn, offers valuable insights into the dietary intervention’s ability to protect against these detrimental conditions. The comet assay has already been implemented in numerous human dietary interventions to assess their protective effects. A recent review summarizing the results of 69 human nutrition-intervention studies in the last 12 years measuring DNA damage via comet assay found that approximately 50% of the studies observed protective effects [41]. Protective effects were most pronounced by studies with certain plant foods and increasing vegetable intake, rather than from individual phytochemicals in supplement form. However, many studies had shortcomings in their study design or statistical analyses. These include not utilizing a cross-over design, baseline tests, or randomization, all of which were addressed in our study design.

In our study, after an overall increase in fruits and vegetables and *ex vivo* exposure of participant lymphocytes to H_2_O_2_, DNA damage decreased by 23% on average, as measured by % tail DNA. Although the tail moment similarly decreased post-intervention, the reduction was not statistically significant. Participants who consumed Blend 1 and Blend 3 were the most protected from DNA damage induced by H_2_O_2_. Older participants tended to have higher DNA damage in general after the *ex vivo* exposure, but they did not respond more positively or negatively to the intervention than the younger participants. Our results are in line with a previous study where 168 participants who consumed 1 L of blueberry–apple juice for 4 weeks showed a 20% protection against *ex vivo*-induced oxidative DNA damage [28]. In a randomized cross-over study with 10 men who consumed 300 g of blended blueberries, H_2_O_2_-induced DNA damage was reduced by 18% 1 h after blueberry consumption compared to a control jelly [43]. Comparing these results with our most similar dietary intervention, Blend 2, which is made mostly of berries, participants who consumed this blend for 2 weeks had a similar (21%) reduction in DNA damage. Even stronger responses were seen after the flavonoid-rich blend containing apples and green tea and the carotenoid-rich blend of carrots, tomato, and red bell pepper (Blend 1 and Blend 3), which resulted in 30% and 32% decreases in *ex vivo*-induced DNA damage, respectively. Our prolonged run-in phase of 2 weeks may have contributed to our larger effect on DNA damage reduction after our F and V interventions, or the specific combination of fruits and vegetables implemented. Contrary to the synergism hypothesis, due to the effect of our F and V mixtures representing a specific class of phytochemicals (Blends 1–4), the effect of the combined blends (Blend 5–7) was not stronger than the effects of their additive benefits on % tail DNA or the tail moment. Blend 6 and Blend 7 resulted in an effect similar to the additive benefits of its component F and V blends, whereas the effect of Blend 5 was reduced compared to its component ingredients, suggesting an antagonistic effect on DNA damage resistance resulting from the blend of phytochemicals in Blend 1 and Blend 2. To control for the effect of overall F and V intake, each blend was portioned to equal 400 g of F and V per day. Therefore, as the blends increased in complexity, the dose of each F and V contributing to the blend was reduced (Table 1), which may also play a role in limiting the protective ability of the more complex blends.

The other marker for DNA damage, investigated at a much smaller scale in this study, was the excretion of oxidized purines (8-OHdG) in urine. This endpoint was intended to serve as a biomarker of systemic DNA damage from naturally occurring DNA oxidation and breakdown. However, contrary to expectations, no change was found in excreted oxidized purines (8-OHdG) after 2 weeks of consuming Blend 7. This assay has been shown to successfully correlate with conditions known to exhibit higher oxidative stress levels, such as in the samples of patients with periodontal disease, Type 2 diabetes, cardiovascular disease, and cancer compared to healthy controls, and it can be predictive of poorer health outcomes in these patients [44,45,46,47]. Levels of oxidized purines have also been positively associated with smoking status and alcohol consumption, and inversely associated with healthy meal combinations, fruit consumption, and daily physical activity [48,49]. An intervention study from 1999 that included 28 women did find a decrease in 8-OHdG levels in urine after an increase of F and V servings from 5.8 to 12 per day, which is roughly 450 g to 975 g per day [49], much higher than what was given in the MiBlend study. Aside from this study and case controls, which indicate differences in levels based on the reported intake [50], there have been no other studies to our knowledge that test the effect of a dietary intervention on 8-OHdG levels in a healthy population. Our findings suggest that a diet sufficient in F and V offers notable benefits during acute episodes of oxidative stress compared to low F and V intake, such as what was replicated in the comet assay, but it does not provide a reduction in naturally oxidized DNA in healthy individuals within a 2-week timespan.

To provide a holistic understanding of how F and V influence oxidative stress beyond its effect on DNA damage, we also measured the antioxidant capacity of plasma and levels of ROS in whole blood. Antioxidant capacity (measured by TEAC) significantly increased in participant plasma after increasing F and V intake from 50 to 450 g/day for 2 weeks. Nearly 90% of antioxidant activity in the human diet is obtained from F and V, with polyphenols as the principal antioxidants, followed by Vitamin C, and then glucosinolate metabolites, Vitamin E, and Vitamin A [51,52]. It has also previously been established that total phenolic content and TEAC values *in vitro* are highly positively correlated. Particularly effective at increasing TEAC levels were Blend 2, Blend 6, and Blend 3. Blend 2 is rich in polyphenols like anthocyanins and led to the highest increase in TEAC [53,54]. Blend 3 and Blend 6 contained F and V with the highest Vitamin C content (e.g., red bell pepper), and participants who consumed these interventions also excreted the highest concentrations of Vitamin C. It follows that these fruit and vegetable combinations would lead to the highest increases in antioxidant capacity. Since these results align with expectations, we can also infer good compliance from our participants.

Conversely, there was no difference in superoxide levels after the intervention despite using spin traps to form stable radical adducts and ESR to detect and quantify their concentration, which is considered the gold standard for measuring free radical concentrations. In a previously conducted randomized controlled trial of 24 participants, reactive oxygen species were measured at 0, 60, 120, and 180 min after ingesting a 100 mg blend of F and V. While the control group did not change in ROS levels from Time 0, the group that consumed F and V significantly decreased, with the greatest reduction at 120 min, which then returned to levels at 180 min similar to that at 60 min. This suggests that measurements of ROS in the blood are best detected shortly after consuming a meal high in F and V, with the peak effect or decrease in ROS occurring around 120 min, after ample time for absorption [55]. This could explain why no difference in ROS was detected at post-intervention versus baseline in our fasting participants.

To assess the ability of F and V to scavenge ROS, it may also be valuable to measure other forms of ROS. In the previously mentioned study, the scavenging of superoxide anion only amounted to 30% of their F and V intervention’s total free radical scavenging activity. A large degree of activity was also seen in reducing peroxyl radicals and hydroxyl radicals. We also have a biological defense system and enzymatic antioxidants that convert superoxide anions into harmless unreactive species. Other ROS like peroxyl radicals, hydroxyl radicals, peroxynitrile, and singlet oxygen are not known to be quenched by endogenous enzymes, so the defense relies on nonenzymatic antioxidants like phytochemicals [56,57]. Therefore, measuring more forms of ROS at around 2 h after consumption may have been ideal for assessing changes in direct ROS scavenging. However, due to the existing complexity of our study, it would have placed an undue burden on the participants to require these additional blood collections.

We also explored if it would be of added value to test lipid peroxidation levels by measuring creatinine-controlled urinary 8-isoprostane concentrations in urine in a small subset of the study population (n = 29), but significant differences were not detectable in post-intervention samples versus baseline. Also, 8-isoprostane is an eicosanoid derived from the nonenzymatic peroxidation of arachidonic acid, catalyzed by free radicals. It is often used as a marker for oxidative stress and lipid peroxidation in the body [58].

A limited number of studies have examined the effects of dietary changes on 8-isoprostane levels in healthy volunteers. In a randomized dietary intervention on 122 premenopausal women with a family history of breast cancer, the authors concluded that it was weight loss that resulted in a reduction in plasma 8-isoprostane levels, not the reduction in dietary fat or increase in F and V intake [59]. A review of 154 interventions testing antioxidant consumption or supplementation on 8-isoprostane changes showed that these dietary antioxidants had less than a 45% success at effectively modulating isoprostane levels [58]. One successful study found 8-isoprostane levels to correlate well with estimated polyphenol intake and excreted polyphenols in urine [60]. Due in part to a limited sample size, we could not confirm the correlation between polyphenol intake and lipid peroxidation levels. Although 8-isoprostane levels appear responsive to antioxidant foods in individuals with oxidative stress-related pathology [57,61,62], its efficacy may also be constrained within our study population due to their health status.

### 4.2. Changes in Retinal Microvasculature

In addition to assessing phenotypic changes in markers related to DNA damage and oxidative stress, we evaluated the interventions’ effect on the retinal microvasculature to enhance our understanding of the role of F and V in preventing cardiovascular disease. This also serves to validate the gene-expression results of previous studies, which showed that increased F and V intake affected pathways related to cardiovascular health [11]. Recent findings from a systematic review, which examined clinical studies spanning the last 2 decades, shed light on the association between retinal microvascular changes (diameter, tortuosity, and branching) and the prevalence or incidence of heart disease in humans. Among the 42 publications analyzed (comprising 14 prospective, 26 cross-sectional, and two retrospective studies), significant associations were observed between retinal vascular changes and various cardiac conditions, including coronary artery disease, heart failure, hypertension, and acute coronary syndrome [63]. Acute coronary syndrome encompasses conditions such as ST-elevation myocardial infarction, non-ST elevation myocardial infarction, and unstable angina. This condition falls under the category of coronary heart disease, accounting for one-third of all deaths in individuals aged 35 and older. Furthermore, retinal vascular diameter (measured by CRAE, CRVE, and AVR), especially in women, correlated with and predicted the incidence of acute coronary syndrome, with the relationship being more pronounced in midlife individuals than in the elderly population. These findings underscore the potential use of retinal imaging as a screening, diagnostic, and prognostic tool for cardiovascular diseases [63].

In our study, consumption of any blend of F and V for 2 weeks had a positive impact on the participants’ vessels, resulting in wider retinal arterioles and narrower venules. Although a significant decrease in venule diameter did occur at Post-test 1, the biggest impact of the increase in F and V was on the arteriolar diameter. Older participants had smaller vessels, so overall smaller venules and arterioles, but did not differ in their response to the intervention compared to the younger participants in the study. Neither branching nor tortuosity changed after the 2-week F and V intervention, which is in line with our expectations with a shorter intervention duration. Male participants had significantly more branching than female participants, but neither sex group saw significant changes in vessel branching after the intervention. Emphasizing whole foods and dietary patterns over individual nutrients appears to be crucial for successfully impacting cardiovascular risk reduction. It seems that a complex interplay of various nutrients may be interacting with genetic factors to influence cardiovascular disease risk. It is worth noting that the dietary interventions (Blend 5 and Blend 1) resulting in the most significant improvements in retinal microvasculature were not the blends highest in expected dietary nitrate or fiber content, factors known to impact vascular health [11,64,65,66,67,68]. This suggests that the effect of these F and V blends may not be solely due to nitrate- or fiber-based mechanisms but rather a combined effort of various phytochemical combinations acting as transcription factors to induce physiological changes.

### 4.3. Absorption and Excretion of Phytochemicals

Although phytochemicals can exhibit antioxidant activity in the intestinal lumen [18], influence the kinetics of other luminal compounds, and interact with the microbiome [69], their absorption in the intestines is crucial for making them available to target tissues and eliciting various systemic effects. Therefore, we measured the phytochemical content of blood samples to confirm the absorption of major classes of these compounds. In this study, no differences were seen in the total polyphenol absorption, while significant increases were seen in carotenoid absorption (especially among those who consumed carotenoid-rich blends), and the excretion of Vitamin C was shown to increase in only the intervention containing the highest expected levels of Vitamin C.

Many polyphenols are rapidly and extensively metabolized in intestinal and liver cells and usually appear as metabolites in the bloodstream before they are excreted in urine. Most reach the small intestine intact, bound to sugar molecules [70]. Studies have shown that conjugated phenolic metabolites appear in the circulatory system within 1–2 h after ingestion of a complex plant capsule, suggesting an initial absorption in the small intestine [69]. Some compounds are conjugated at both the enterocyte and the hepatic level before entering the systemic circulatory system. A concentration peak was also recorded between 5 and 10 h after ingestion, which suggests an interaction between indigested polyphenol fractions and colonic microbiota, resulting in low molecular weight compounds, which are efficiently reabsorbed by colonocytes before hepatic conjugation [69]. Plasma levels were shown to drop in polyphenol concentration 10 h after intake. As we sampled blood in our study after 12 h of fasting, this may explain why we did not find higher concentrations of polyphenols in plasma after the intervention phases. Also, *in vitro* analyses showed that polyphenols can bind to albumin, the most abundant plasma protein, which was removed before the analysis of total polyphenols during deproteinization, potentially contributing further to the lack of differences detected after the intervention [71].

Carotenoids are fat-soluble and are, therefore, better absorbed when consumed in combination with dietary fat, and this was not controlled for in the study. The interventions themselves contained negligible amounts of dietary fat, but participants were encouraged to consume their blends with their meals. In enterocytes, some Vitamin A precursors are cleaved and converted into retinol or retinoic acid, depending on Vitamin A status. Retinoic acid is transported bound to albumin via the portal vein to the liver [72], whereas retinol and the uncleaved carotenoids are incorporated into chylomicrons, transported throughout the lymphatic system, released into the bloodstream, and then taken up by the liver, where they are either cleaved or incorporated into circulating lipoproteins to be deposited into various tissues [72]. Lutein is stored in the macula where it absorbs blue light to optimize visual function [73], lycopene accumulates in the liver, adrenal glands, testes, and skin [74], while alpha- and beta-carotene are stored in the liver and adipose tissues [75]. Alpha- and Beta-carotene are precursors to Vitamin A (retinol), which is necessary for maintaining healthy vision, skin, and immune system [75]. Retinol can be converted to retinoic acid to regulate gene expression, influencing numerous physiological processes.

Carotenoids’ half-life can range from several days to a few weeks, depending on intake, genetics, and overall health [73,74,75]. Their longer absorption is likely why it was detected best of the measured phytochemicals. Lutein, alpha-carotene, and beta-carotene increased in the plasma of all participants after the 2-week increase in F and V, but not lycopene. This may be explained by the fact that lycopene is present in much smaller amounts in the various F and V mixes. Post-test 2 did not show a dose-responsive increase from Post-test 1, confirming that the 1-week washout period was sufficient to eliminate a confounding effect for this measure. Beta-carotene levels did not differ between males and females at baseline, but females did have significantly higher levels post-intervention compared with males. The increase in beta-carotene levels in females was not enough to show a significant difference compared with their baseline but does suggest that females may be slightly more responsive to an increase in plasma beta-carotene levels after an F and V intervention.

Based on the ingredient composition of the blends and previously measured phytochemical content of these ingredients [29], Blend 3 and Blend 7 should contain the highest levels of lutein (Supplementary Figure S1), and a subsequent increase in plasma lutein levels after consuming those blends was confirmed. Participant plasma levels of alpha- and beta-carotene evaluated by intervention-group also follow expectations, as Blend 3, Blend 7, and then Blend 6 should contain the highest levels of these carotenoids.

Participants who consumed Blend 3, which should contain the highest amount of Vitamin C of all blends, had significantly higher concentrations of excreted Vitamin C after that intervention. The blend that should contain the next highest level of Vitamin C, Blend 4, did, in fact, show a similar increase in mean excreted Vitamin C among the participants who consumed this intervention, but due to the small sample size, this could not be determined as statistically significant. It should be noted that Vitamin C levels were measured in urine after many months in −80 °C storage. As Vitamin C degrades over time in cold storage, the analytical model corrected for this effect, which explained 9.1% of the variance in Vitamin C measurements.

Inter-individual variations in the concentrations of phytochemicals in blood and tissues have been linked to genetic differences, particularly arising from related single nucleotide polymorphisms. A randomized controlled trial has shown that some genetic variants related to phytochemical absorption (quercetin, Vitamin C) can impact their absorption levels and are worth investigating further [28]. Future work is planned to investigate the effects of these genotypic differences in the MiBLEND study. This, plus expected absorption peaks, chemical half-lives, and stability, are all worth considering when aiming to measure phytochemical absorption following an F and V intervention.

### 4.4. Gene-Expression Changes and Pathway Analysis

Gene-expression analysis was performed to gain insights into potential mechanisms behind F and V’s role in disease prevention. The lower number of DEGs for each intervention group’s post-test versus baseline expression values is due to the smaller group sizes (n = ~40), compared to the number of DEGs derived from the group comparing all post-test values versus all baseline values in the “All DIs” (“All Blends”) comparison.

Pathway analysis was performed on the 846 significant genes, which were differentially expressed after an overall increase in F and V for 2 weeks in 121 participants. In order of significance (*q* value), the first overrepresented pathway was “HDACs deacetylate histones”, then “RNA Polymerase I promoter opening”, “Recognition and association of DNA glycosylase with site containing an affected purine”, “Cleavage of the damaged purine”, “PRC2 methylates histones and DNA”, “RHO GTPase cycle”, “Depurination”, “RNA Polymerase I promoter escape”, “Deposition of new CENPA-containing nucleosomes at the centromere”, and, finally, “Nucleosome assembly”. These pathways relate to changes in the cell cycle, chromatin organization, gene expression, DNA repair, and signal transduction.

Histone deacetylases (HDACs) are enzymes that play a crucial role in the regulation of gene expression and chromatin structure. They are involved in the dynamic process of modifying histone proteins, which are key components of chromatin, the complex of DNA and proteins that makeup chromosomes. HDACs remove acetyl groups from lysine residues on histone proteins, leading to a more condensed chromatin structure and often repressing gene transcription. Based on the directionality of the significant DEGs in this group (Supplementary Table S2), we can infer that since histone deacetylation is being down-regulated with the increase in F and V intake, this potentially allows for the transcription of otherwise inaccessible genes. Dietary flavonoids have been associated with epigenetic effects, particularly in modulating DNA methylation and histone acetylation, holding promise as a target for cancer prevention [76,77]. The link between phytochemical intake and HDAC inhibition will be further discussed in the context of the GSEA analysis results below.

The pathway “RNA Polymerase I promoter opening” is involved in the initiation of transcription by RNA Polymerase I (Pol I), where the DNA helix is unwound at the promotor region so that Pol I can bind to the promoter and initiate ribosomal RNA transcription [78]. The “RNA Polymerase I promoter clearance” pathway represents when Pol I transitions from the promoter region of the DNA strand to moving along the DNA template [78]. While not significant in the Reactome pathway analysis, “RNA Polymerase I Promoter Escape” was significantly under-represented after all blends in the GSEA analysis, which represents Pol I’s successful clearance of the promoter to the chain elongation phase of transcription [78]. A recent study utilizing *in vitro* and animal models found that a Pol I inhibitor resulted in partial reversal of pulmonary arterial hypertension, prevention of perivascular inflammation, and activation of the tumor suppressor p53 [79]. This suggests that phytochemicals might influence transcription factors, subsequently affecting rRNA gene transcription and ribosome biogenesis, leading to disease-preventative effects similar to those shown in a separate study after inhibiting Pol I *in vivo*. The under-represented pathway “PRC2 methylates histones and DNA” also suggests an alteration of the chromatin structure to induce gene expression.

“Depurination” involves the spontaneous loss of a purine base from a nucleotide in DNA resulting in an apurinic (AP) site (i.e., abasic site), often triggering DNA repair mechanisms81. Depurination can occur naturally due to spontaneous hydrolysis or other endogenous factors, chemical reactions, exposure to oxidative stress via free radicals, or environmental exposure to damaging or mutagenic agents. Alternatively, damaged or mismatched purines that were not spontaneously removed must first be recognized by a DNA glycosylase in order to initiate DNA repair [78]. This pathway “recognition and association of DNA glycosylase with site containing an affected purine” represents this step in the “base excision repair” (BER) pathway. Then, the subsequent “cleavage of damaged purine” pathway involves the recognition and removal of the damaged or mismatched purine base by the DNA glycosylase enzyme [78]. The under-representation detected in these pathways suggests that after 2 weeks of a phytochemical-rich diet, DNA repair mechanisms were in less demand, potentially due to the increased antioxidant capacity of their plasma.

The “RHO GTPase cycle” pathway refers to the dynamic process of activation and inactivation of Rho family GTPases, a class of small signaling proteins that play a critical role in regulating various cellular processes, including cell migration, adhesion, and proliferation, as well as cytoskeletal organization and gene expression. Rho GTPases are part of the larger Ras superfamily of GTPases and act as molecular switches that relay signals from cell-surface receptors to downstream cellular machinery [79]. Changes in gene expression in this pathway likely have consequences relating to altered downstream signaling cascades. Rho GTPases dysregulation has been associated with diseases such as cancer and cardiovascular disorders [79,80]. Therefore, changes in gene expression within the Rho GTPase pathways could have implications for disease prevention or progression. A 2019 review by Tsakiroglou et al. summarized the research linking anthocyanins and other polyphenols with the activities of Rho GTPases, particularly endothelial cell migration and angiogenesis [81]. Similar to our pathway analysis and GSEA results, other studies saw consistent inhibition of angiogenesis and cell migration from exposure to anthocyanins and quercetin *in vitro*, *in vivo*, and *ex vivo*, suggesting benefits against conditions like atherosclerosis and tumor growth [81]. The specific consequences of these alterations, of course, depend on the nature and context of the gene expression changes, as well as the specific Rho GTPases involved.

The “nucleosome assembly” pathway involves the process of assembling nucleosomes to ensure proper packaging of DNA and regulating access to the underlying genetic information [38]. The “Deposition of new CENPA-containing nucleosomes at the centromere” pathway involves incorporating CENP-A histone variants at centromere regions of chromosomes to create specialized centromeric nucleosomes that establish centromere identity and function. Proper CENP-A deposition is essential for centromere function, accurate chromosome segregation, and the attachment of spindle microtubules during cell division [38]. Alterations in the nucleosome assembly pathway after an increase in fruit and vegetable intake could have implications for gene regulation, genomic stability, cellular differentiation, and responses to environmental stimuli. As this is a novel finding, specific mechanisms would need to be investigated further to fully understand the biological significance of these changes in the context of health outcomes.

GSEA analysis was performed for each F and V blend and again for the overall increase in F and V to detect more subtle changes in gene-expression pathways compared to baseline. GSEA considers sets of genes that share common biological functions or are involved in the same pathways in contrast to DEG analysis, which focuses on the individual genes whose change in expression surpasses a designated threshold. Analyzing gene sets provides a more holistic view of the biological processes that might be impacted by each F and V blend, which could not have been found with pathway analysis on the few significant DEGs per intervention group. GSEA is also more sensitive to subtle but coordinated changes in gene expression across a pathway. Even if the changes in individual genes are modest, GSEA can detect collective shifts in the expression of gene sets, making it more powerful in capturing biologically relevant changes. GSEA is also more robust in handling sample heterogeneity and small effect sizes. Similar to the previous pathway analysis, the results of the GSEA showed that when combining all interventions, changes in expression of biological pathways after a 400 g/day increase in F and V intake mostly involve cell cycle, immune system, signal transduction, gene expression, and DNA repair pathways. The majority of the pathways altered after each specific blend of F and V also belonged to these categories (Figure 8).

The change in HDAC expression seen with all blends compared to the baseline in the DEG analysis and subsequent pathway analysis was also seen in the GSEA analysis for the participants who had Blends 1, 2, 3, 4, and 5. The GSEA results also showed under-enrichment of the “HATs acetylate histones” pathway, which generally leads to a more condensed chromatin structure and less accessibility of DNA to the transcriptional machinery, repressing gene expression. The depression of both of these pathways suggests more complex changes in epigenetic regulation and gene expression, which should be further explored.

Some phytochemicals are known to act as HDAC inhibitors, which means they can block the activity of HDAC enzymes and promote histone acetylation. This inhibition of HDACs can result in altered gene-expression patterns and has been linked to various biological effects, including anti-cancer and anti-inflammatory properties. In particular, resveratrol (present particularly in Blend 2 and Blend 5), sulforaphane (an isothiocyanate present in Blend 4), and epigallocatechin gallate (a catechin particularly present in Blend 1 and Blend 5) have been studied for their potential effect in histone deacetylation inhibition or histone acetylation [82,83,84,85,86]. *In vitro* analyses found that resveratrol inhibited all 11 human HDACs of Classes I, II, and IV in a dose-dependent manner. HDACs of Classes I, II, and IV are involved in cancer development or progression and inhibitors of HDACs are being investigated as promising anticancer drugs. Resveratrol was demonstrated to also have HDAC inhibitory activity *ex vivo* on human blood samples [85]. Epigallocatechin gallate, a major green tea polyphenol, has been shown *in vitro* to act as an HDAC inhibitor in cellular and cell-free models. The results of a recent *in vitro* trial indicated that epigallocatechin gallate promotes chromatin relaxation in human endothelial cells and presents broad epigenetic potential affecting the expression and activity of epigenome modulators [86]. Isothiocyanates have also been found to inhibit HDAC expression and/or activity in cancer cells, mouse models, and in human studies [15,82,83,84,87,88,89,90]. Therefore, this could explain the reduction in histone deacetylase expression after the consumption of Blends 1–5.

Another common theme in observed gene-expression changes were in pathways involved in signal transduction. Particularly Blend 1, Blend 3, and Blend 4 resulted in downward enrichment of pathways involving signaling by receptor tyrosine kinases and RAF/MAP kinase cascades. Flavonoids (rich in Blend 1) and carotenoids (rich in Blend 3) have been studied for their ability to affect cell signaling or bind to transcription factors, as these signals can mediate tumor progression. Findings from cell-culture experiments indicate that many of the effects of flavonoids, such as their anti-inflammatory, antidiabetic, anticancer, and neuroprotective activities, are associated with their capacity to influence cell-signaling pathways [91]. Notably, the intracellular concentrations of flavonoids required to modulate cellular signaling are significantly lower than those needed to impact cellular antioxidant capacity. Furthermore, even if their antioxidant activity diminishes, flavonoid metabolites may still maintain their ability to interact with cell-signaling proteins [91,92,93]. Numerous *in vitro* studies suggest that flavonoids may influence chronic diseases by selectively inhibiting kinases [91,94]. Moreover, cell growth and proliferation are regulated by growth factors that initiate cell-signaling cascades by binding to specific receptors on cell membranes. Flavonoids might modulate growth-factor signaling by inhibiting receptor phosphorylation or by blocking the binding of growth factors to their receptors [95]. We found receptor tyrosine kinases to be upwardly enriched in our results, which is not unexpected in a healthy population and suggests a regulatory role of flavonoids on kinase regulation.

In our study, the carotenoid-rich F and V blend (Blend 3) led to downward enrichment of MAP kinase activation and signaling cascades, and previous studies have also shown that carotenoids can affect similar pathways. Treatments involving astaxanthin, α-carotene, lycopene, siphonoxanthin, and β-carotene have been associated with significantly decreased levels of phosphorylated Akt, JNK, p38, and ERK1/2, the most characterized subfamilies of MAPKs [96]. Since MAPK family proteins play vital roles in activating NF-κB and triggering cellular events that promote the enhanced survival of cancer cells, these findings highlight the role of carotenoids in cancer prevention.

Aside from gene expression, cell cycle, and DNA-replication pathways, the other major pathway with reduced expression after each blend was DNA repair, suggesting a major effect of increased phytochemical consumption that resulted in a decreased need for DNA repair processes. Phytochemicals present in our blends have also been shown to have DNA repair or protection activities in other studies. Catechins, which are present in the green tea mixed into Blend 1 and Blends 5–7, have been previously shown to trigger redox-sensitive cytoprotective adaptations. After both a single dose and regular intake of green tea, the activity of the DNA repair enzyme human oxoguanine glycosylase 1 increased in participant lymphocytes [97]. Carotenoids (notably present in Blend 3, Blend 6, and Blend 7) have been shown to modulate DNA damage by repairing the balance between DNA damage and repair in human lymphocytes. Dietary supplementation with cooked carrots increased the repair activity of oxidized purines, improving repair patch synthesis activity in participant lymphocytes [98]. Compounds in Blend 4 and Blend 7 (isothiocyanates) have been found to modulate the activity of Phase I biotransformation enzymes, especially those of the cytochrome P450 (CYP) family in animal experiments [99,100]. Some carcinogens require Phase I enzymes in order to become active carcinogens capable of binding DNA and forming cancer-causing DNA adducts, so the inhibition of specific CYP enzymes may reduce cancer development in animal models. Isothiocyanates, derived from the hydrolysis of glucosinolates, are potent inducers of Phase II detoxifying enzymes that protect cells from DNA damage by carcinogens and ROS [101]. Limited data from clinical studies have shown that the ingestion of foods high in glucosinolates/isothiocyanates leads to increased UGT activity, which is critical for the biotransformation and elimination of harmful compounds and increased plasma and intestinal glutathione S-transferase, a critical endogenous antioxidant and detoxicant enzyme [102,103,104]. The findings from our transcriptomic analysis, coupled with insights from prior studies affirming the DNA-protective properties of phytochemicals, are further substantiated by our comet assay results. These results reveal that participant lymphocytes, subjected to *ex vivo* oxidative stress following the F and V intervention, demonstrate enhanced protection against DNA damage.

Participants who consumed Blend 3 exhibited downregulation in pathways associated with lipoprotein clearance, including pathways such as “Plasma lipoprotein clearance” (NES = −1.69), “plasma lipoprotein assembly, remodeling, and clearance” (NES = −1.54), and “VLDLR internalization and degradation” (NES = −1.65). This finding is particularly relevant because carotenoids, known for their high-fat solubility and low-water solubility, circulate within lipoproteins, sharing this transport mechanism with cholesterol and other fats. The correlation between low-density lipoprotein (LDL) oxidation and atherosclerosis development prompted scientists to explore the role of antioxidant compounds, such as carotenoids, in the prevention of cardiovascular disease [105]. Interestingly, HDL-cholesterol concentration was positively linked to serum α-carotene, β-cryptoxanthin, and lutein/zeaxanthin concentrations, with the latter exhibiting an inverse association with LDL-cholesterol, which are important risk factors for cardiovascular disease [106]. Numerous case-control and cross-sectional studies have identified higher blood concentrations of carotenoids as significantly associated with reduced measures of carotid artery intima-media thickness [107,108,109,110,111,112]. This thickness is a reliable marker of atherosclerosis [113].

Some limitations of our study and future recommendations relate to our study population characteristics. It is possible that the participants who responded to recruitment flyers for the study were also those who were more health-conscious. In order to compare more effectively the potential differences in responses among males and females, evenly matched sex groups would be ideal. However, we were still able to note some differences in characteristics in our 30% male population and otherwise control for sex-related differences in order to provide general conclusions. Also of note, the significant difference in weight at post-tests compared to baseline did not exist in an intermediate analysis prior to the COVID-19 pandemic. We suspect that global changes in lifestyle had a larger impact on the modest weight gain observed in this study. Although the pandemic was not a controllable force, we did control for related immune system alterations by excluding sick participants and not allowing participants to join the study until 2 weeks after recovery from an infection or vaccine administration. The statistically significant but clinically small increase in nutrients from participant food diaries is only partially explained by the dietary intervention, suggesting that during intervention phases, participants replaced some nutrients habitually consumed at baseline with their F and V intervention. This was assessed by comparing the average increase in nutrients with the “Average Blend” nutrient information in Table 3, which was calculated using the weighted average of interventions completed from food diary data (which was blinded to the participants). However, in line with the small increase in nutrient intake at intervention phases, the nutrient replacement was also minor. Perhaps the form that the F and V were administered in (essentially a “smoothie” format) encouraged participants to view the interventions as supplementary, rather than something that takes up space on the meal plate. Future interventions aimed at encouraging healthy lifestyle changes could improve the displacement of less healthful foods by F and V by promoting less processed plant food integration in the diet. One important component of the blends that we did not investigate explicitly was the fiber content. The role that adequate fiber plays in glucose and lipid metabolism, in gut–brain axis health, on microbiome composition, and in small-chain fatty-acid formation cannot be ignored as a potential contributor to the effects seen here. Although too complex for this study, future studies could look deeper into the phytochemical–fiber relationship of whole plant foods. Additionally, while adequate fiber intake is linked with improved carbohydrate metabolism and chronic disease prevention, in future interventions, a weekly gradual increase to these higher fiber levels is optimal to avoid potential gastrointestinal disturbances. The study duration of 2 weeks was long enough to detect changes in gene-expression levels after a significant increase in F and V intake, but the duration is, of course, not long enough to see long-term clinical improvements, especially not in a healthy population. Performing a human dietary intervention of this scale in an at-risk population and in a long-term study could glean additional valuable insights.

## 5. Conclusions

The results of our study demonstrated that increasing fruit and vegetable intake to the levels advised by the WHO improved protection against DNA damage, increased antioxidant capacity of plasma, and improved retinal microvascularization. These dietary changes also altered gene expression in pathways relating to DNA repair, cell cycle, transcription, replication, immune response, and signal transduction after only 2 weeks of intervention, thus identifying crucial mechanisms that are potentially involved in disease prevention. Blend 1, a combination of apples and green tea, was among the best at reducing susceptibility to DNA damage and improving overall microvasculature (AVR). Blend 3, the carotenoid blend of carrots, tomato, and bell pepper was the most effective at reducing DNA damage susceptibility and increasing the antioxidant capacity of plasma. Blend 5, a mix of Blend 1 with berries and blue grapes, and Blend 7, the most complex blend of polyphenols, carotenoids, and glucosinolates, were also very effective at improving overall AVR. Also of note, Blend 2, the anthocyanin-rich blend of blueberries, raspberries, blackberries, and red grape, and Blend 6, a blend of F and V from Blend 1, Blend 2, and Blend 3, also significantly improved antioxidant capacity. Overall, one blend did not outperform all others for each endpoint. Therefore, while a general increase in non-starchy fruits and vegetables can improve many different markers of chronic disease risk, tailoring disease-prevention strategies to an individual’s unique health profile and risks may be supported by recommending a particular blend of fruits and vegetables.

## Figures and Tables

**Table 1:** Dietary intervention compositions.

Blend	Composition	Over-Represented Phytochemicals	Fruits and Vegetables	Calories (kcal)	Carbohydrate (g)	Fat (g)	Protein (g)	Sodium (mg)	Sugars (g)	Fiber (g)
1	1	Flavonoids (e.g., quercetin, catechins)	Apples (400 g), green tea (2 g in 100 mL water ^a^)	207	55	1	1	4	41	10
2	2	Anthocyanins (subclass of flavonoids)	Blueberries (100 g), blue grapes (100 g), blackberries (100 g), raspberries (100 g)	222	54	1	3	3	34	14
3	3	Carotenoids	Tomato (133 g), carrots (133 g), red bell pepper (133 g)	112	26	1	4	103	15	8
4	4	Diallyl sulfide Glucosinulates	Broccoli (133 g), cauliflower (133 g), Brussels sprouts (133 g)	136	27	1	11	117	8	11
5	1 + 2	Flavonoids Anthocyanins	Apples (200 g), green tea (1 g in 50 mL water), blueberries (50 g),blue grapes (50 g), blackberries (50 g), raspberries (50 g)	218	54	1	3	3	36	13
6	1 + 2 + 3	Flavonoids Anthocyanins Carotenoids	Apples (133 g), green tea (0.66 g in 33 mL water), blueberries (33 g), blue grape (33 g), blackberries (33 g), raspberries (33 g), tomato (44 g), carrots (44 g), red bell pepper (44 g)	178	44	1	3	41	28	11
7	1 + 2 + 3 + 4 (most complex mixture)	Flavonoids Anthocyanins Carotenoids Diallyl sulfide Glucosinulates	Apples (100 g), green tea (0.5 g in 25 mL water), blueberries (25 g), blue grape (25 g), blackberries (25 g), raspberries (25 g), tomato (33 g), carrots (33 g), red bell pepper (33 g), broccoli (33 g), cauliflower (33 g), Brussels sprouts (33 g)	176	39	1	5	62	23	11

^a^ Water was boiled and subsequently cooled for 2 min. Subsequently, 2 g of green tea were added to 100 mL water and steeped for 2 min, after which the green tea was removed [30].

**Table 4:** Number of significant DEGs after FDR correction from 10,577 genes and listed by intervention group. All groups are comparisons made by the intervention group’s post-test samples with their matched baseline samples.

	Blend 1	Blend 2	Blend 3	Blend 4	Blend 5	Blend 6	Blend 7	All Blends
Down-regulated	0	1	1	18	0	9	3	519
Not Significant	10,576	10,575	10,575	10,559	10,577	10,568	10,573	9731
Up-regulated	1	1	1	0	0	0	1	327

**Table 5:** Overview of the significantly overrepresented pathways after an increased intake of 450 g/day F and V for 2 weeks compared to 50 g/day for 2 weeks.

Pathway Category	Pathway Name	*q* Value	# of Entities	Total in Pathway
Cell Cycle	Deposition of new CENPA-containing nucleosomes at the centromere	4.65 × 10^−2^	10	53
	Nucleosome assembly	4.65 × 10^−2^	10	53
Chromatin Organization	HDACs deacetylate histones	3.17 × 10^−6^	19	63
DNA Repair	Recognition and association of DNA glycosylase with site containing an affected purine	1.94 × 10^−2^	10	38
	Cleavage of the damaged purine	2.20 × 10^−2^	10	44
	Depurination	2.69 × 10^−2^	10	45
Gene Expression (Transcription)	RNA Polymerase I Promoter Opening	1.78 × 10^−2^	10	33
	PRC2 methylates histones and DNA	2.20 × 10^−2^	10	43
	RNA Polymerase I Promoter Escape	3.18 × 10^−2^	12	60
Signal Transduction	RHO GTPase cycle	2.21 × 10^−2^	44	460

## Data Availability

Some data described in the manuscript, code book, and analytic code will be made publicly and freely available without restriction at https://github.com/jndeben/miblend, accessed on 29 March 2024. The raw datasets presented in this article are not readily available because the data are part of an ongoing study. Requests to access the datasets should be directed to s.vanbreda@maastrichtuniversity.nl.

## Supplementary Materials

The following supporting information can be downloaded at: https://www.mdpi.com/article/10.3390/antiox13080915/s1, Figure S1: The phytochemical content in the ingredients, Table S1: Number of participants per group by assay, Figure S2: Vitamin C levels by test day and duration in cold storage, Table S2: DEGs found in significantly overrepresented pathways in Reactome for all blends versus baseline comparison, Table S3: GSEA-enriched pathways after 2 weeks of consuming 400 g/day of Blend 1. NES = normalized enrichment score, Table S4: GSEA-enriched pathways after 2 weeks of consuming 400 g/day of Blend 2. NES = normalized enrichment score, Table S5: GSEA-enriched pathways after 2 weeks of consuming 400 g/day of Blend 3. NES = normalized enrichment score, Table S6: GSEA-enriched pathways after 2 weeks of consuming 400 g/day of Blend 4. NES = normalized enrichment score, Table S7: GSEA-enriched pathways after 2 weeks of consuming 400 g/day of Blend 5. NES = normalized enrichment score, Table S8: GSEA-enriched pathways after 2 weeks of consuming 400 g/day of Blend 6. NES = normalized enrichment score, Table S9: GSEA-enriched pathways after 2 weeks of consuming 400 g/day of Blend 7. NES = normalized enrichment score, Table S10: GSEA-enriched pathways after 2 weeks of consuming 400 g F and V per day (all blends). NES = normalized enrichment score.

## References

[1] Riboli E., Norat T. (2003). Epidemiologic evidence of the protective effect of fruit and vegetables on cancer ris-4. Am. J. Clin. Nutr..

[2] Aune D., Keum N., Giovannucci E., Fadnes L.T., Boffetta P., Greenwood D.C., Tonstad S., Vatten L.J., Riboli E., Norat T. (2018). Dietary intake and blood concentrations of antioxidants and the risk of cardiovascular disease, total cancer, and all-cause mortality: A systematic review and dose-response meta-analysis of prospective studies. Am. J. Clin. Nutr..

[3] Aune D., Chan D.S.M., Vieira A.R., Navarro Rosenblatt D.A., Vieira R., Greenwood D.C., Norat T. (2017). Fruit and vegetable intake and the risk of cardiovascular disease, total cancer and all-cause mortality-A systematic review and dose-response meta-analysis of prospective studies. Int. J. Epidemiol..

[4] Amine E.K., Baba N.H., Belhadj M., Deurenberg-Yap M., Djazayery A., Forrestre T., Galuska D.A., Herman S., James W.P.T., M’Buyamba Kabangu J.R. (2003). Diet, Nutrition and the Prevention of Chronic Diseases.

[5] RIVM (2020). Eet en drinkt Nederland volgens de richtlijnen Schijf van Vijf?: Resultaten van de Voedselconsumptiepeiling, 2012–2016.

[6] Eurostat (2022). How Much Fruit and Vegetables Do You Eat Daily? Europa.eu. https://ec.europa.eu/eurostat/web/products-eurostat-news/-/ddn-20220104-1.

[7] Lee S., Moore L., Park S., Harris D., Blanck H. (2022). Adults Meeting Fruit and Vegetable Intake Recommendations—United States, 2019. Morb. Mortal. Wkly. Rep..

[8] Scalbert A., Andres-Lacueva C., Arita M., Kroon P., Manach C., Urpi-Sarda M., Wishart D. (2011). Databases on Food Phytochemicals and Their Health-Promoting Effects. J. Agric. Food Chem..

[9] van Breda S.G.J., de Kok T.M.C.M. (2018). Smart Combinations of Bioactive Compounds in Fruits and Vegetables May Guide New Strategies for Personalized Prevention of Chronic Diseases. Mol. Nutr. Food Res..

[10] van Breda S.G.J., Wilms L.C., Gaj S., Jennen D.G.J., Briedé J.J., Kleinjans J.C.S., De Kok T.M.C.M. (2015). The exposome concept in a human nutrigenomics study: Evaluating the impact of exposure to a complex mixture of phytochemicals using transcriptomics signatures. Mutagenesis.

[11] van Breda S.G.J., Wilms L.C., Gaj S., Jennen D.G.J., Briedé J.J., Helsper J.P., Kleinjans J.C.S., De Kok T.M.C.M. (2014). Can Transcriptomics Provide Insight into the Chemopreventive Mechanisms of Complex Mixtures of Phytochemicals in Humans?. Antioxid. Redox Signal..

[12] Chang J.L., Chen G., Ulrich C.M., Bigler J., King I.B., Schwarz Y., Li S., Li L., Potter J.D., Lampe J.W. (2010). DNA Damage and Repair: Fruit and Vegetable Effects in a Feeding Trial. Nutr. Cancer.

[13] González-Vallinas M., González-Castejón M., Rodríguez-Casado A., Ramírez De Molina A. (2013). Dietary phytochemicals in cancer prevention and therapy: A complementary approach with promising perspectives. Nutr. Rev..

[14] Saracino M.R., Lampe J.W. (2007). Phytochemical Regulation of UDP-Glucuronosyltransferases: Implications for Cancer Prevention. Nutr. Cancer.

[15] Dashwood R.H., Ho E. (2007). Dietary histone deacetylase inhibitors: From cells to mice to man. Semin. Cancer Biol..

[16] Hecht S.S. (1999). Symposium on Phytochemicals: Biochemistry and Physiology Chemoprevention of Cancer by Isothiocyanates, Modifiers of Carcinogen Metabolism. J. Nutr..

[17] Barcelo S., Gardiner J.M., Gescher A., Chipman J.K. (1996). CYP2E1-mediated mechanism of anti-genotoxicity of the broccoli constituent sulforaphane. Carcinogenesis.

[18] van Breda S.G., Mathijs K., Pieters H.-J., Sági-Kiss V., Kuhnle G.G., Georgiadis P., Saccani G., Parolari G., Virgili R., Sinha R. (2021). Replacement of Nitrite in Meat Products by Natural Bioactive Compounds Results in Reduced Exposure to N-Nitroso Compounds: The PHYTOME Project. Mol. Nutr. Food Res..

[19] Wallace T.C., Bailey R.L., Blumberg J.B., Burton-Freeman B., Chen C., Crowe-White K.M., Drewnowski A., Hooshmand S., Johnson E., Lewis R. (2020). Fruits, vegetables, and health: A comprehensive narrative, umbrella review of the science and recommendations for enhanced public policy to improve intake. Crit. Rev. Food Sci. Nutr..

[20] Michalska M., Gluba A., Mikhailidis D.P., Nowak P., Bielecka-Dabrowa A., Rysz J., Banach M. (2010). The role of polyphenols in cardiovascular disease. Med. Sci. Monit..

[21] Murphy K.J., Chronopoulos A.K., Singh I., Francis M.A., Moriarty H., Pike M.J., Turner A.H., Mann N.J., Sinclair A.J. (2003). Dietary flavanols and procyanidin oligomers from cocoa (Theobroma cacao) inhibit platelet function. Am. J. Clin. Nutr..

[22] Mukai Y., Sato S. (2009). Polyphenol-containing azuki bean (Vigna angularis) extract attenuates blood pressure elevation and modulates nitric oxide synthase and caveolin-1 expressions in rats with hypertension. Nutr. Metab. Cardiovasc. Dis..

[23] Fisher N.D.L., Hughes M., Gerhard-Herman M., Hollenberg N.K. (2003). Flavanol-rich cocoa induces nitric-oxide-dependent vasodilation in healthy humans. J. Hypertens..

[24] Surh Y.-J. (2004). Transcription Factors in the Cellular Signaling Network as Prime Targets of Chemopreventive Phytochemicals. Cancer Res. Treat. Off. J. Korean Cancer Assoc..

[25] Khan F., Niaz K., Maqbool F., Ismail Hassan F., Abdollahi M., Nagulapalli Venkata K.C., Nabavi S.M., Bishayee A. (2016). Molecular Targets Underlying the Anticancer Effects of Quercetin: An Update. Nutrients.

[26] van Breda S.G.J., van Agen E., Engels L.G., Moonen E.J., Kleinjans J.C., van Delft J.H. (2004). Altered vegetable intake affects pivotal carcinogenesis pathways in colon mucosa from adenoma patients and controls. Carcinogenesis.

[27] Thompson H.J., Heimendinger J., Diker A., O’Neill C., Haegele A., Meinecke B., Wolfe P., Sedlacek S., Zhu Z., Jiang W. (2006). Dietary Botanical Diversity Affects the Reduction of Oxidative Biomarkers in Women due to High Vegetable and Fruit Intake. J. Nutr..

[28] Wilms L.C., Boots A.W., de Boer V.C., Maas L.M., Pachen D.M., Gottschalk R.W., Ketelslegers H.B., Godschalk R.W., Haenen G.R., van Schooten F.J. (2007). Impact of multiple genetic polymorphisms on effects of a 4-week blueberry juice intervention on ex vivo induced lymphocytic DNA damage in human volunteers. Carcinogenesis.

[29] DeBenedictis J.N., de Kok T.M., van Breda S.G. (2023). Impact of Processing Method and Storage Time on Phytochemical Concentrations in an Antioxidant-Rich Food Mixture. Antioxidants.

[30] Vermeer I.T.M., Moonen E.J.C., Dallinga J.W., Kleinjans J.C.S., Van Maanen J.M.S. (1999). Effect of ascorbic acid and green tea on endogenous formation of N-nitrosodimethylamine and N-nitrosopiperidine in humans. Mutat. Res. Fundam. Mol. Mech. Mutagen..

[31] Mrakic-Sposta S., Gussoni M., Montorsi M., Porcelli S., Vezzoli A. (2012). Assessment of a Standardized ROS Production Profile in Humans by Electron Paramagnetic Resonance. Oxid. Med. Cell Longev..

[32] Van Breda S.G.J., Briedé J.J., De Kok T.M.C.M. (2018). Improved Preventive Effects of Combined Bioactive Compounds Present in Different Blueberry Varieties as Compared to Single Phytochemicals. Nutrients.

[33] Dams S., Holasek S., Tsiountsioura M., Malliga D.E., Meier-Allard N., Poncza B., Lackner S., Jansenberger Y., Lamprecht M. (2021). An encapsulated fruit, vegetable and berry juice powder concentrate increases plasma values of specific carotenoids and vitamins. Int. J. Vitam. Nutr. Res..

[34] Verheijen M., Sarkans U., Wolski W., Jennen D., Caiment F., Kleinjans J. (2022). HeCaToS Consortium. Multi-omics HeCatoS dataset of repeated dose toxicity for cardiotoxic; hepatotoxic compounds. Sci. Data.

[35] Verheijen M.C., Meier M.J., Asensio J.O., Gant T.W., Tong W., Yauk C.L., Caiment F. (2022). R-ODAF: Omics data analysis framework for regulatory application. Regul. Toxicol. Pharmacol..

[36] Robinson M.D., Oshlack A. (2010). A scaling normalization method for differential expression analysis of RNA-seq data. Genome Biol..

[37] Law C.W., Alhamdoosh M., Su S., Dong X., Tian L., Smyth G.K., Ritchie M.E. (2016). RNA-seq analysis is easy as 1-2-3 with limma, Glimma and edgeR. F1000Res.

[38] Fabregat A., Sidiropoulos K., Viteri G., Forner O., Marin-Garcia P., Arnau V., D’Eustachio P., Stein L., Hermjakob H. (2017). Reactome pathway analysis: A high-performance in-memory approach. BMC Bioinform..

[39] Page G.P., Edwards J.W., Gadbury G.L., Yelisetti P., Wang J., Trivedi P., Allison D.B. (2006). The PowerAtlas: A power and sample size atlas for microarray experimental design and research. BMC Bioinform..

[40] Altman D.G., Bland J.M. (1999). How to randomize. BMJ.

[41] Mišík M., Staudinger M., Kundi M., Worel N., Nersesyan A., Ferk F., Dusinska M., Azqueta A., Møller P., Knasmueller S. (2023). Use of the single cell gel electrophoresis assay for the detection of DNA-protective dietary factors: Results of human intervention studies. Mutat. Res. Rev. Mutat. Res..

[42] Nandhakumar S., Parasuraman S., Shanmugam M.M., Rao K.R., Chand P., Bhat B.V. (2011). Evaluation of DNA damage using single-cell gel electrophoresis (Comet Assay). J. Pharmacol. Pharmacother..

[43] Del Bó C., Riso P., Campolo J., Møller P., Loft S., Klimis-Zacas D., Brambilla A., Rizzolo A., Porrini M. (2013). A single portion of blueberry (Vaccinium corymbosum L) improves protection against DNA damage but not vascular function in healthy male volunteers. Nutr. Res..

[44] Paredes-Sánchez E., Montiel-Company J.M., Iranzo-Cortés J.E., Almerich-Torres T., Bellot-Arcís C., Almerich-Silla J.M. (2018). Meta-Analysis of the Use of 8-OHdG in Saliva as a Marker of Periodontal Disease. Dis. Markers.

[45] Nishikawa T., Sasahara T., Kiritoshi S., Sonoda K., Senokuchi T., Matsuo T., Kukidome D., Wake N., Matsumura T., Miyamura N. (2003). Evaluation of urinary 8-hydroxydeoxy-guanosine as a novel biomarker of macrovascular complications in type 2 diabetes. Diabetes Care.

[46] Ersöz A., Diltemiz S.E., Özcan A.A., Denizli A., Say R. (2008). Synergie between molecular imprinted polymer based on solid-phase extraction and quartz crystal microbalance technique for 8-OHdG sensing. Biosens. Bioelectron..

[47] Kroese L.J., Scheffer P.G. (2014). 8-Hydroxy-2′-Deoxyguanosine and Cardiovascular Disease: A Systematic Review. Curr. Atheroscler. Rep..

[48] Tamae K., Kawai K., Yamasaki S., Kawanami K., Ikeda M., Takahashi K., Miyamoto T., Kato N., Kasai H. (2009). Effect of age, smoking and other lifestyle factors on urinary 7-methylguanine and 8-hydroxydeoxyguanosine. Cancer Sci..

[49] Thompson H.J., Heimendinger J., Haegele A., Sedlacek S.M., Gillette C., O’Neill C., Wolfe P., Conry C. (1999). Effect of increased vegetable and fruit consumption on markers of oxidative cellular damage. Carcinogenesis.

[50] Edalati S., Bagherzadeh F., Jafarabadi M.A., Ebrahimi-Mamaghani M. (2021). Higher ultra-processed food intake is associated with higher DNA damage in healthy adolescents. Br. J. Nutr..

[51] Landete J.M. (2013). Dietary Intake of Natural Antioxidants: Vitamins and Polyphenols. Crit. Rev. Food Sci. Nutr..

[52] Scalbert A., Johnson I.T., Saltmarsh M. (2005). Polyphenols: Antioxidants and beyond. Am. J. Clin. Nutr..

[53] Shan B., Cai Y.Z., Sun M., Corke H. (2005). Antioxidant Capacity of 26 Spice Extracts and Characterization of Their Phenolic Constituents. J. Agric. Food Chem..

[54] Chohan M., Naughton D.P., Jones L., Opara E.I. (2012). An Investigation of the Relationship between the Anti-Inflammatory Activity, Polyphenolic Content, and Antioxidant Activities of Cooked and In Vitro Digested Culinary Herbs. Oxid. Med. Cell Longev..

[55] Nemzer B., Chang T., Xie Z., Pietrzkowski Z., Reyes T., Ou B. (2014). Decrease of free radical concentrations in humans following consumption of a high antioxidant capacity natural product. Food Sci. Nutr..

[56] Higdon J.V., Frei B. (2003). Tea Catechins and Polyphenols: Health Effects, Metabolism, and Antioxidant Functions. Crit. Rev. Food Sci. Nutr..

[57] Dhawan V., Jain S. (2004). Effect of garlic supplementation on oxidized low density lipoproteins and lipid peroxidation in patients of essential hypertension. Mol. Cell Biochem..

[58] Petrosino T., Serafini M. (2014). Antioxidant Modulation of F2-Isoprostanes in Humans: A Systematic Review. Crit. Rev. Food Sci. Nutr..

[59] Chen G., Heilbrun L.K., Venkatramanamoorthy R., Maranci V., Redd J.N., Klurfeld D.M., Djuric Z. (2004). Effects of low-fat and/or high fruit-and-vegetable diets on plasma levels of 8-isoprostane-F2α in the nutrition and breast health study. Nutr. Cancer.

[60] Ruiz N., Segarra A.B., Lara L., Ramírez-Sánchez M., Prieto I. (2019). Diet and Oxidative Status. The Dietary Pattern and Urinary 8-Isoprostane in Healthy Spanish Women. Antioxidants.

[61] Santus P., Sola A., Carlucci P., Fumagalli F., Di Gennaro A., Mondoni M., Carnini C., Centanni S., Sala A. (2005). Lipid peroxidation and 5-lipoxygenase activity in chronic obstructive pulmonary disease. Am. J. Respir. Crit. Care Med..

[62] Serafini M., Miglio C., Peluso I., Petrosino T. (2011). Modulation of Plasma Non Enzimatic Antioxidant Capacity (NEAC) by Plant Foods: The Role of Polyphenol. Curr. Top. Med. Chem..

[63] Allon R., Aronov M., Belkin M., Maor E., Shechter M., Fabian I.D. (2021). Retinal Microvascular Signs as Screening and Prognostic Factors for Cardiac Disease: A Systematic Review of Current Evidence. Am. J. Med..

[64] McCall D.O., McGartland C.P., McKinley M.C., Patterson C.C., Sharpe P., McCance D.R., Young I.S., Woodside J.V. (2009). Dietary intake of fruits and vegetables improves microvascular function in hypertensive subjects in a dose-dependent manner. Circulation.

[65] Kapil V., Webb A.J., Ahluwalia A. (2010). Inorganic nitrate and the cardiovascular system. Heart.

[66] Crilly M.A., Mcneill G. (2012). Arterial dysfunction in patients with rheumatoid arthritis and the consumption of daily fruits and daily vegetables. Eur. J. Clin. Nutr..

[67] Gopinath B., Liew G., Lewis J.R., Blekkenhorst L.C., Bondonno C., Burlutsky G., Hodgson J.M., Mitchell P. (2020). Association of dietary nitrate intake with retinal microvascular structure in older adults. Eur. J. Nutr..

[68] Kan H., Stevens J., Heiss G., Klein R., Rose K.M., London S.J. (2007). Dietary fiber intake and retinal vascular caliber in the Atherosclerosis Risk in Communities Study 1-3. Am. J. Clin. Nutr..

[69] Bresciani L., Martini D., Mena P., Tassotti M., Calani L., Brigati G., Brighenti F., Holasek S., Malliga D.E., Lamprecht M. (2017). Absorption profile of (Poly)phenolic compounds after consumption of three food supplements containing 36 different fruits, vegetables, and berries. Nutrients.

[70] Gonzales G.B., Smagghe G., Grootaert C., Zotti M., Raes K., Van Camp J. (2015). Flavonoid interactions during digestion, absorption, distribution and metabolism: A sequential structure-activity/property relationship-based approach in the study of bioavailability and bioactivity. Drug Metab. Rev..

[71] Latruffe N., Menzel M., Delmas D., Buchet R., Lançon A. (2014). Compared Binding Properties between Resveratrol and Other Polyphenols to Plasmatic Albumin: Consequences for the Health Protecting Effect of Dietary Plant Microcomponents. Molecules.

[72] Wang X. (2014). Carotenoids.

[73] Krinsky N.I., Landrum J.T., Bone R.A. (2003). Biologic mechanisms of the protective role of lutein and zeaxanthin in the eye. Annu. Rev. Nutr..

[74] Moran N.E., Erdman J.W.B., Clinton S.K. (2013). Complex interactions between dietary and genetic factors impact lycopene metabolism and distribution. Arch. Biochem. Biophys..

[75] Gropper S., Smith J., Yolanda C. (2013). Advanced Nutrition and Human Metabolism.

[76] Jiang W., Xia T., Liu C., Li J., Zhang W., Sun C. (2021). Remodeling the Epigenetic Landscape of Cancer—Application Potential of Flavonoids in the Prevention and Treatment of Cancer. Front. Oncol..

[77] Busch C., Burkard M., Leischner C., Lauer U.M., Frank J., Venturelli S. (2015). Epigenetic activities of flavonoids in the prevention and treatment of cancer. Clin. Epigenetics.

[78] Xu X., Feng H., Dai C., Lu W., Zhang J., Guo X., Yin Q., Wang J., Cui X., Jiang F. (2021). Therapeutic efficacy of the novel selective RNA polymerase I inhibitor CX-5461 on pulmonary arterial hypertension and associated vascular remodelling. Br. J. Pharmacol..

[79] Olson M.F. (2018). Rho GTPases, their post-translational modifications, disease-associated mutations and pharmacological inhibitors. Small GTPases.

[80] Haga R.B., Ridley A.J. (2016). Rho GTPases: Regulation and roles in cancer cell biology. Small GTPases.

[81] Tsakiroglou P., VandenAkker N.E., Del B.Ó.C., Riso P., Klimis-Zacas D. (2019). Role of Berry Anthocyanins and Phenolic Acids on Cell Migration and Angiogenesis: An Updated Overview. Nutrients.

[82] Myzak M.C., Tong P., Dashwood W.-M., Dashwood R.H., Ho E. (2007). Sulforaphane Retards the Growth of Human PC-3 Xenografts and Inhibits HDAC Activity in Human Subjects. Exp. Biol. Med..

[83] Rajendran P., Delage B., Dashwood W.M., Yu T.W., Wuth B., Williams D.E., Ho E., Dashwood R.H. (2011). Histone deacetylase turnover and recovery in sulforaphane-treated colon cancer cells: Competing actions of 14-3-3 and Pin1 in HDAC3/SMRT corepressor complex dissociation/reassembly. Mol. Cancer.

[84] Rajendran P., Delage B., Dashwood W.M., Yu T.W., Wuth B., Williams D.E., Ho E., Dashwood R.H. (2013). HDAC turnover, CtIP acetylation and dysregulated DNA damage signaling in colon cancer cells treated with sulforaphane and related dietary isothiocyanates. Epigenetics.

[85] Venturelli S., Berger A., Bö Cker A., Busch C., Weiland T. (2013). Resveratrol as a Pan-HDAC Inhibitor Alters the Acetylation Status of Jistone Proteins in Human-Derived Hepatoblastoma Cells. PLoS ONE.

[86] Ciesielski O., Biesiekierska M., Balcerczyk A. (2020). Epigallocatechin-3-gallate (EGCG) Alters Histone Acetylation and Methylation and Impacts Chromatin Architecture Profile in Human Endothelial Cells. Molecules.

[87] Abbaoui B., Telu K.H., Lucas C.R., Thomas-Ahner J.M., Schwartz S.J., Clinton S.K., Freitas M.A., Mortazavi A. (2017). The impact of cruciferous vegetable isothiocyanates on histone acetylation and histone phosphorylation in bladder cancer. J. Proteom..

[88] Batra S., Sahu R.P., Kandala P.K., Srivastava S.K. (2010). Benzyl isothiocyanate-mediated inhibition of histone deacetylase leads to NF-kappaB turnoff in human pancreatic carcinoma cells. Mol. Cancer Ther..

[89] Clarke J.D., Hsu A., Yu Z., Dashwood R.H., Ho E. (2011). Differential effects of sulforaphane on histone deacetylases, cell cycle arrest and apoptosis in normal prostate cells versus hyperplastic and cancerous prostate cells. Mol. Nutr. Food Res..

[90] Pledgie-Tracy A., Sobolewski M.D., Davidson N.E. (2007). Sulforaphane induces cell type-specific apoptosis in human breast cancer cell lines. Mol. Cancer Ther..

[91] Williams R.J., Spencer J.P.E., Rice-Evans C. (2004). Flavonoids: Antioxidants or signalling molecules?. Free Radic. Biol. Med..

[92] Spencer J.P.E., Schroeter H., Rechner A.R., Rice-Evans C. (2004). Bioavailability of Flavan-3-ols and Procyanidins: Gastrointestinal Tract Influences and Their Relevance to Bioactive Forms In Vivo. Antioxid. Redox Signal..

[93] Spencer J.P.E. (2003). Metabolism of tea flavonoids in the gastrointestinal tract. J. Nutr..

[94] Hou D.-X., Fujii M., Terahara N., Yoshimoto M. (2004). Molecular Mechanisms Behind the Chemopreventive Effects of Anthocyanidins. J. Biomed. Biotechnol..

[95] Lambert J.D., Yang C.S. (2003). Mechanisms of cancer prevention by tea constituents. J. Nutr..

[96] Kumar Saini R., Keum Y.-S., Daglia M., Rengasamy K.R. (2020). Dietary carotenoids in cancer chemoprevention and chemotherapy: A review of emerging evidence. Pharmacol. Res..

[97] Ho C.K., Choi S.-W., Siu P.M., Benzie I.F.F. (2014). Effects of single dose and regular intake of green tea (Camellia sinensis) on DNA damage, DNA repair, and heme oxygenase-1 expression in a randomized controlled human supplementation study. Mol. Nutr. Food Res..

[98] Astley S.B., Elliott R.M., Archer D.B., Southon S. (2004). Evidence that dietary supplementation with carotenoids and carotenoid-rich foods modulates the DNA damage:repair balance in human lymphocytes. Br. J. Nutr..

[99] La Marca M., Beffy P., Della Croce C., Gervasi P.G., Iori R., Puccinelli E., Longo V. (2012). Structural influence of isothiocyanates on expression of cytochrome P450, phase II enzymes, and activation of Nrf2 in primary rat hepatocytes. Food Chem. Toxicol..

[100] Ioannides C., Konsue N. (2015). A principal mechanism for the cancer chemopreventive activity of phenethyl isothiocyanate is modulation of carcinogen metabolism. Drug Metab. Rev..

[101] Kensler T.W., Talalay P. (2004). Inducers of Enzymes That Protect Against Carcinogens and Oxidants. Cancer Chemoprevention.

[102] Von Weymarn L.B., Chun J.A., Hollenberg P.F. (2006). Effects of benzyl and phenethyl isothiocyanate on P450s 2A6 and 2A13: Potential for chemoprevention in smokers. Carcinogenesis.

[103] Nijhoff W.A., Grubben M.J., Nagengast F.M., Jansen J.B., Verhagen H., van Poppel G., Peters W.H. (1995). Effects of consumption of Brussels sprouts on intestinal and lymphocytic glutathione S-transferases in humans. Carcinogenesis.

[104] Nijhoff W.A., Mulder T.P.J., Verhagen H., Van Poppel G., Peters W.H.M. (1995). Effects of consumption of brussels sprouts on plasma and urinary glutathione S-transferase class-alpha and -pi in humans. Carcinogenesis.

[105] Kritchevsky S.B. (1999). Recent Advances in Nutritional Science-Carotene, Carotenoids and the Prevention of Coronary Heart Disease 1. J. Nutr..

[106] Wang Y., Chung S.J., McCullough M.L., Song W.O., Fernandez M.L., Koo S.I., Chun O.K. (2014). Dietary carotenoids are associated with cardiovascular disease risk biomarkers mediated by serum carotenoid concentrations. J. Nutr..

[107] McQuillan B.M., Hung J., Beilby J.P., Nidorf M., Thompson P.L. (2001). Antioxidant vitamins and the risk of carotid atherosclerosis: The perth carotid ultrasound disease assessment study (CUDAS). J. Am. Coll. Cardiol..

[108] Iribarren C., Folsom A.R., Jacobs DRJr Gross M.D., Belcher J.D., Eckfeldt J.H. (1997). Association of Serum Vitamin Levels, LDL Susceptibility to Oxidation, and Autoantibodies Against MDA-LDL With Carotid Atherosclerosis. Arterioscler. Thromb. Vasc. Biol..

[109] D’Odorico A., Martines D., Kiechl S., Egger G., Oberhollenzer F., Bonvicini P., Sturniolo G.C., Naccarato R., Willeit J. (2000). High plasma levels of alpha- and beta-carotene are associated with a lower risk of atherosclerosis: Results from the Bruneck study. Atherosclerosis.

[110] Rissanen T., Voutilainen S., Nyyssönen K., Salonen R., Salonen J.T. (2000). Low Plasma Lycopene Concentration Is Associated With Increased Intima-Media Thickness of the Carotid Artery Wall. Arterioscler. Thromb. Vasc. Biol..

[111] Dwyer J.H., Paul-Labrador M.J., Fan J., Shircore A.M., Merz C.N., Dwyer K.M. (2004). Progression of Carotid Intima-Media Thickness and Plasma Antioxidants: The Los Angeles Atherosclerosis Study. Arterioscler. Thromb. Vasc. Biol..

[112] Rissanen T.H., Voutilainen S., Nyyssönen K., Salonen R., Kaplan G.A., Salonen J.T. (2003). Serum lycopene concentrations and carotid atherosclerosis: The Kuopio Ischaemic Heart Disease Risk Factor Study. Am. J. Clin. Nutr..

[113] Bots M.L., Grobbee D.E. (2002). Intima Media Thickness as a Surrogate Marker for Generalised Atherosclerosis. Cardiovasc. Drugs Ther..

